# Comparative evaluation of insulin resistance indices for predicting adverse cardiovascular events in hyperuricemia: insights from UK biobank and Shanghai Pudong cohort

**DOI:** 10.3389/fnut.2026.1870901

**Published:** 2026-07-13

**Authors:** Honglin Liu, Shuhao Cui, Lijun Chang, Yan Wu, Xiongbo Liang, Wentao Shi, Jinjin Liu, Yang Yang, Yuan Liu

**Affiliations:** 1 Department of Medical Records, The Affiliated Hospital of Qingdao University, Qingdao, China; 2 Department of Clinical Laboratory, Zhongshan Second People's Hospital, Zhongshan, China; 3 Department of Orthopaedics, Peking University Third Hospital, Beijing, China; 4 School of Public Health, Shanghai Jiao Tong University School of Medicine, Shanghai, China; 5 Clinical Research Unit, Shanghai Ninth People’s Hospital, Shanghai Jiao Tong University School of Medicine, Shanghai, China; 6 Zhabei Central Hospital of Jing'an District, Shanghai, China

**Keywords:** adverse cardiovascular events, hyperuricemia, inflammatory markers, insulin resistance-related indices, Mendelian randomization, phenotypic aging

## Abstract

**Background:**

Hyperuricemia (HUA) frequently coexists with glucolipid metabolic disturbances characterized by insulin resistance, dyslipidemia, and adipose tissue dysfunction. However, the association between insulin resistance (IR)-related indices and cardiovascular disease (CVD) incidence/mortality in HUA patients remains not yet fully established.

**Methods:**

Data from the UK Biobank (UKBB) and Shanghai Pudong cohort were analyzed, enrolling 35,853 and 12,680 HUA patients, respectively. Associations between seven IR-related indices and adverse cardiovascular events were assessed using multivariate Cox proportional hazards models, restricted cubic spline analyses, time-dependent receiver operating characteristic, random forest modeling, and Mendelian randomization. Mediation analyses evaluated the role of phenotypic aging biomarkers and inflammatory markers in these relationships.

**Results:**

All seven IR indices exhibited significant associations with CVD events and mortality in HUA patients from two cohorts. TyG-WHTR showed comparable 10-year CVD mortality predictive performance to SCORE2 in UKBB (AUC, 0.733). Elevated TyG-WHTR correlated with increased CVD mortality [T3 vs. T1: HR, 1.76 (95%CI: 1.48–2.11)], while higher CMI predicted myocardial infarction [HR, 1.52 (1.31–1.76)]. C-reactive protein (mediation ratio: 1.3–13.3%) and accelerated aging (4.5–34.4%) partially mediated these associations. Genetically predicted TyG index elevation was linked to myocardial infarction risk [OR, 1.79 (1.48–2.16)].

**Conclusion:**

IR-related indices serve as clinically valuable tools for CVD risk stratification in HUA, with inflammatory activation and accelerated aging constituting key mechanistic pathways. These findings provide novel insights for early identification and targeted prevention of cardiovascular complications in HUA patients.

## 1 Introduction

Cardiovascular diseases (CVD) persist as the predominant contributor to global mortality and disability-adjusted life years. This health burden has escalated disproportionately in low and middle-income countries, with cardiovascular deaths projected to reach 35.6 million in 2050 (1). Several investigations have revealed that cardiovascular events arise from complex interactions between inflammatory cascades and metabolic dysregulation (2). Hyperuricemia (HUA), defined by sustained serum urate elevation, constitutes a cardinal feature of metabolic syndrome (3). Beyond its established association with gout pathogenesis, HUA independently potentiates CVD risk through multifaceted mechanisms including pro-inflammatory cytokine activation, endothelial homeostasis disruption, vascular remodeling stimulation, and renin-angiotensin-aldosterone system upregulation (4). Concurrent with global shifts in lifestyle and diet, epidemiological trends indicate a progressive rise in HUA prevalence, estimated 13.85% in 2025 (5). Due to cardiovascular complications emerging as critical determinants of morbidity and functional impairment, there remains an urgent need to develop risk-stratification frameworks for early CVD detection in HUA.

Insulin resistance (IR) represents a pathophysiological condition characterized by diminished target tissue responsiveness to insulin. Mechanistically connected to cardiovascular pathogenesis, IR potentiates CVD progression through chronic low-grade inflammation and dysregulated lipid homeostasis (6). Clinically validated composite biomarkers have emerged as reliable indicators for IR quantification and cardiometabolic risk stratification, including the triglyceride-glucose (TyG) index, triglyceride-to-high-density lipoprotein cholesterol (TG/HDL-C) ratio, metabolic score for insulin resistance (METS-IR), and cardiometabolic index (CMI) (7–9). Notably, HUA and IR exhibit bidirectional crosstalk through overlapping metabolic pathways, synergistically amplifying cardiovascular risks (10). Nevertheless, robust longitudinal evidence remains scarce regarding the prognostic efficacy of these indices for CVD prediction specifically in HUA populations.

Aging represents an irreversible biological phenomenon marked by multisystem functional deterioration, manifesting hallmark phenotypes including immune senescence, cellular aging, and telomere attrition (11). This progressive process substantially elevates susceptibility to chronic diseases and cardiovascular mortality (12), while maintaining intricate associations with metabolic dysregulation. IR, recognized as a cardinal metabolic feature of aging, demonstrates significantly higher prevalence in geriatric populations (13). Under IR conditions, persistent hyperglycemia and dyslipidemia induce oxidative stress and chronic inflammation, subsequently driving DNA damage, accelerated vascular endothelial senescence, and atherogenesis progression (14, 15). Emerging evidence from longitudinal cohort studies underscores that established biological aging metrics, particularly phenotypic age and biological age, mediate the associations between IR and both all-cause and cardiovascular mortality (16). Notably, uric acid exerts dual pathological effects within the IR network. HUA amplifies inflammatory cascades via NLRP3 inflammasome activation, thereby establishing a self-perpetuating cycle with IR progression (17). However, critical knowledge gaps persist regarding whether biomarkers like phenotypic age and C-reactive protein (CRP) mediate relationships between IR indices and adverse cardiovascular outcomes in HUA populations. Furthermore, the relative contributions of distinct pathological pathways involving inflammatory signaling, phenotypic regulation, and telomeric damage mechanisms remain to be elucidated through well-designed cohorts.

In this study, we leverage data from the UK Biobank (UKBB), Shanghai Pudong cohort and publicly accessible genome-wide association study (GWAS) repositories to achieve four principal objectives: (1) systematically evaluate associations between seven IR-related indices and cardiovascular outcomes in HUA; (2) conduct cross-population validation of identified relationships; (3) investigate potential mediation effects through phenotypic aging biomarkers and inflammatory cascades; (4) provide supportive evidence for potential causal associations between the TyG index and CVD outcomes using Mendelian randomization. Collectively, we aim to advance preventive strategies for hyperuricemia-associated cardiovascular morbidity.

## 2 Methods

### 2.1 Study design and study population

This study utilized data from two independent cohorts: the Pudong cohort and the UK Biobank (UKBB 2006–2010). The UKBB component comprises a population-based prospective cohort study conducted between 2006 and 2010 across England, Wales, and Scotland, encompassing comprehensive lifestyle records and health parameters from over 500,000 participants aged 40–69 years. After applying exclusion criteria for missing hematological profiles, incomplete physical examination records, or insufficient outcome follow-up data, we identified 35,853 qualified HUA cases for subsequent analysis (Supplementary Figure S1). Recruitment for the Shanghai Pudong Cohort was conducted in three waves between 2019 and 2021. Following exclusion of participants with missing data, 12,680 cases of hyperuricemia were retained for the final analysis (Supplementary Figure S2).

### 2.2 Definition of seven IR-related indices

We chose seven insulin resistance indices: TyG, TyG-body mass index (TyG-BMI), TyG-waist circumference (TyG-WC), TyG-waist-to-height ratio (TyG-WHTR), triglyceride-to-high-density lipoprotein cholesterol (TG/ HDL-C) ratio, metabolic score for insulin resistance (METS-IR) and cardiometabolic index (CMI). Seven insulin resistance-related indices employed in this study have been previously validated against the gold-standard hyperinsulinemic-euglycemic clamp (7, 9, 18, 19). The calculation formulas are as follows:

TyG index = Ln[Triglyceride (mg/dL) × Glucose (mg/dL)/2];

TyG-BMI = TyG × BMI;

TyG-WC = TyG × Waist Circumference;

TyG-WHTR = TyG × WHTR = TyG × Waist Circumference/Height;

TG/HDL-C = Triglycerides (mg/dL)/HDL-C (mg/dL);

METS-IR = Ln[2 × Fasting blood glucose (mg/dL) + Fasting triglycerides (mg/dL)] × BMI (kg/m^2^)/Ln[HDL-C (mg/dL)];

CMI, [TG (mmol/L)/HDL-C (mmol/L)] × Waist Circumference/Height (8, 20, 21).

### 2.3 Ascertainment of HUA

HUA was defined according to sex-specific thresholds: serum uric acid levels >7.0 mg/dL (420 μmol/L) for males and >6.0 mg/dL (360 μmol/L) for females, consistent with established clinical criteria (22). In UKBB and Shanghai Pudong Cohort, HUA status was determined through serum uric acid measurements derived from standardized blood biochemical analyses.

### 2.4 Definition of adverse cardiovascular events

The study’s primary endpoints included all-cause mortality, cardiovascular disease (CVD) mortality, and CVD incidence. Mortality status was ascertained through linkage with authoritative death registries: national death notifications for the UKBB. In the UKBB, incident CVD was defined by ICD-10 codes for myocardial infarction (MI) (I21-I22) and ischemic stroke (I63–I64), corroborated by hospital records and clinical imaging. The outcome in the Shanghai Pudong cohort was all-cause mortality, with mortality data obtained from the death registry of the Pudong New Area Center for Disease Control and Prevention. Follow-up was completed on December 31, 2024.

### 2.5 Definition of phenotypic aging and inflammatory markers

In the UKBB datasets, neutrophil count, leukocyte count, monocyte count, and CRP were measured using automated hematology analyzers. These inflammatory indices were treated as continuous variables in our mediation analysis models. The Z-standardized relative leukocyte telomere length,an aging-related biomarker, was directly extracted from the UKBB dataset. Phenotypic age was developed based on the mortality risk of clinical biomarkers and chronological age. To assess phenotypic age acceleration, we employed linear regression to compute residuals of phenotypic age after adjusting for chronological age. Participants with positive residuals (residual > 0) were classified as having an older-than-real phenotypic age, whereas those with negative residuals (residual < 0) were categorized as having a younger-than-real phenotypic age. The formula of phenotypic age is as follows (23):

$\text{phenotypic}\;\mathrm{age}=141.50225+\frac{\text{In}[\begin{array}{l} -0.00553\times \\ \text{In}(1-\text{mortality risk}) \end{array}]}{0.09016.}$

$\text{mortality risk}=1–e^{-e^{\mathrm{xb}}[\exp(120\times \gamma )-1]/\gamma }$

γ=0.0076927

xb=−19.907−0.0336×albumin(g/L)

+0.0095×creatinine(umol/L)+0.1953×glucose(mmol/L)

+0.0954×ln[CRP(mg/L)]−0.012×lymphocyte percentage

$\begin{array}{l} +0.0268\times \text{mean corpuscular volume}\\ +0.3306\times \mathrm{red}\;\text{cell distribution width} \end{array}$

$\begin{array}{l} +0.00188\times \text{alkaline phosphatase}\;(U/L)\\ +0.0554\times \text{white blood cell count} \end{array}$

+0.0804×chronologicalage.

### 2.6 Covariates

In both cohorts, covariates were adjusted for continuous age, sex (male/female), and race (UKBB: White, Asian or Asian British, Black or Black British, Chinese, Mixed, Other ethnic group). Common adjustments also included educational level (less than high school/high school and above), smoking status (yes/no), physical activity level (adequate/inadequate), history of diabetes, and continuous total cholesterol (TC). Alcohol consumption was treated as a continuous variable in the UKBB, whereas it was categorized into a binary drinking status (yes/no) in the Shanghai Pudong Cohort. In the UKBB cohort, continuous alcohol consumption was retained, and the Townsend deprivation index was included as a categorical variable stratified into three tertile-based categories.

### 2.7 Mendelian randomization and sensitivity analysis

Two-sample MR analyses were conducted in European, East Asian and mixed European-East Asian populations, and the data used were derived from publicly available large-scale GWAS summary datasets. Genetic variation in the TyG index was obtained from the previous GWAS study based on the UK Biobank cohort^1^, (24); genetic data on blood biochemical parameters (triglycerides, serum uric acid, etc.) were obtained from the Integrative Epidemiology Unit (IEU) OpenGWAS database, and data on adverse cardiovascular events were integrated from the IEU OpenGWAS database^2^ and FinnGen (freeze 12)^3^. All included studies were approved by the respective institutional review boards and ethics committees. For the mixed population, we screened for single nucleotide polymorphisms (SNPs) as instrumental variables at the genome-wide significance level (*p* < 5 × 10^−8^) and filtered by linkage disequilibrium (*R^2^* < 0.001, within 10,000 kb) to eliminate potential correlational interference. For European and East Asian populations, we relaxed the genome-wide significance criterion (*p* < 5 × 10^−6^) and adjusted the linkage disequilibrium threshold (*R^2^* < 0.01, within 5,000 kb) in order to obtain sufficient instrumental variables, consistent with MR best practices for underpowered GWAS (25, 26). The F-statistic was calculated to remove SNPs with *F* < 10. The inverse variance weighting (IVW) method was used as the main analytical tool, and MR-Egger regression and weighted median method were used as supplementary analyses to verify the robustness of the results. Heterogeneity of SNP effect estimates was assessed by Cochran’s Q statistic and level multicollinearity was tested with the help of MR-Egger regression. MR-PRESSO analysis was adopted for global pleiotropy testing and outlier SNP detection. Steiger directional test was used to verify the overall causal direction, and Steiger filtering was performed simultaneously to eliminate all SNPs whose explanatory power for the outcome exceeded that for the exposure.

### 2.8 Statistical analyses

Independent samples *t*-tests and Pearson’s chi-square tests were used to compare baseline demographic characteristics across study populations. Continuous variables are reported as mean ± standard deviation (SD), while categorical variables are presented as frequency (percentage, %).

First, we employed multivariate Cox proportional hazards models and logistic regression models to explore the associations between TyG, TyG-BMI, TyG-WC, TyG-WHTR, TG/HDL-C, METS-IR, CMI and adverse cardiovascular events. Following adjusting the corresponding covariates, we retained the estimated hazard ratios (HRs) with 95% confidence intervals (CIs) from Cox models and odds ratios (ORs) with 95% CIs from logistic regression, thereby enabling clear visualization of quantitative associations. The Cox proportional hazards model, which adheres to the proportional hazards assumption, was used to estimate survival probabilities within a discrete-time framework as previously described (27).

Subsequently, restricted cubic spline (RCS) curves were employed to investigate the nonlinear associations between exposure indicators and outcomes. As demonstrated in prior research (28), the RCS model serves as an effective tool for modeling nonlinear relationships between continuous variables and health outcomes, enabling flexible estimation of dose–response patterns.

Additionally, we examined the mediating roles of biochemical indicators and phenotypic age acceleration. Mediation analyses were performed using the causal mediation analysis function in SAS under a counterfactual-based framework for survival outcomes. Each mediator was evaluated in a separate model. Traditional mediation analysis quantifies effects through estimation of three components: (1) total effect-the influence of the independent variable on the outcome via all possible pathways; (2) direct effect-the residual association between exposure and outcome, i.e., the effect excluding mediating pathways; (3) indirect effect-the pathway-specific influence of exposure on outcomes transmitted through mediators. When these three effects exhibit consistent directional associations, a “mediation ratio” can be calculated, defined as the proportion of the indirect effect relative to the total effect (29).

The prediction accuracy of each indicator in the random forest plot was evaluated using the receiver operating characteristic curve (ROC), which was drawn using the “randomForest” and “pROC” software package of R software. The dataset was randomly split into a training set (70%) and a test set (30%) at a ratio of 7:3. The training set was used for model development, and the independent test set was applied to evaluate model performance and plot ROC curves. A random seed was set to ensure reproducibility. For sensitivity analysis, 5-fold cross-validation was additionally conducted to verify the robustness and stability of model performance. The entire dataset was randomly divided into 5 non-overlapping subsets. In each iteration, four subsets were used for model training, and the remaining one subset was applied for validation. This process was repeated 5 times until all subsets were validated once. The ROC curve was constructed based on the predicted probabilities, and the AUC was calculated to assess the discriminative ability. For the random forest models, the predictors included IR-related indices and covariates used in the multivariable models, whereas outcome variables and follow-up information were not included as predictors to avoid outcome leakage. TimeROC curve was used to validate the accuracy of prediction models. We used time-dependent ROC curves to assess the ability of seven parameters to predict mortality, with time cut-offs of 120 months. To verify the predictive performance of IR-related indices, we also analyzed the predictive performance of SCORE2 (Europe) and PREVENT equations (America) for the corresponding population (30, 31). Specific details of the SCORE2 (Europe) and PREVENT equations (America) are given in Supplementary Tables S1, S2.

Subgroup analyses were performed by stratifying the total population based on age and sex to assess potential interaction effects of these demographic factors on the associations between exposures and outcomes. Additionally, to validate the robustness of our findings, exposure variables in the UKBB cohort were stratified into quartiles for sensitivity analysis. To evaluate residual confounding, an additional sensitivity analysis was conducted by further adjusting for LDL-C, histories of hypertension and diabetes, and medication use (antihypertensives, glucose-lowering agents, and lipid-lowering drugs). To account for potential bias from competing events (e.g., non-CVD mortality), we performed sensitivity analyses using the Fine-Gray subdistribution hazard model to estimate subdistribution hazard ratios (sHRs).

Statistical analyses for the Shanghai Pudong cohort and UKBB were conducted using R (version 4.4.1) and SAS (version 9.4). Mendelian randomization (MR) analyses were performed in R (version 4.4.2) using the “TwoSampleMR,” “MR-PRESSO,” and “Forestploter” packages. A two-sided significance threshold of *p* < 0.05 was applied across all statistical tests.

## 3 Results

### 3.1 Baseline characteristics of participants

Table 1 presents baseline characteristics of 35,853 UKBB participants with HUA. Between the HUA group and the non-HUA group, all baseline characteristics differed significantly except for educational levels. Compared with the non-HUA group, individuals with HUA were older on average, more likely to be male, and had a higher proportion of low-income earners. Additionally, nearly all anthropometric and biochemical indicators were elevated in the HUA group, with the exception of HDL-C. Conversely, the non-HUA group demonstrated higher rates of non-smoking, non-drinking, and adequate activity, along with higher HDL-C levels. In the Shanghai Pudong cohort, individuals with HUA were younger and predominantly female, with higher rates of drinking and adequate physical activity. They also had lower smoking rate, decreased TC, TyG and METS-IR levels, and a lower mortality burden. During the follow-up period (mean = 1982.5 days, SD = 307.0), 1,162 deaths occurred in the HUA group.

**Table 1:** Baseline characteristics of participants from UK Biobank.

Characteristic	UK Biobank		*p-*value
	(*N* = 252,974)	(*N* = 35,853)	
With hyperuricemia	No	Yes	
Age, mean (SD), years	56.1 (8.1)	57.4 (7.8)	<0.001
Male, *n* (%)	112,865 (44.6)	22,832 (63.7)	<0.001
White, *n* (%)	240,970 (95.3)	34,068 (95.0)	0.049
Less than high school, *n* (%)	68,724 (27.2)	9,770 (27.3)	0.744
Low household income^a^, *n* (%)	42,490 (16.8)	6,789 (18.9)	<0.001
Non-smoking, *n* (%)	140,938 (55.7)	17,168 (47.9)	<0.001
Non-drinking, *n* (%)	22,106 (8.7)	2,704 (7.5)	<0.001
Adequate activity, *n* (%)	192,218 (76.0)	26,426 (73.7)	<0.001
Height, mean (SD), cm	169 (9.2)	171 (9.4)	<0.001
BMI, mean (SD), kg/m^2^	26.7 (4.3)	30.2 (5.0)	<0.001
WC, mean (SD), cm	88.4 (12.7)	99.3 (12.2)	<0.001
Uric acid, mean (SD), umol/L	290 (63.1)	442 (51.1)	<0.001
TC, mean (SD), mg/dL	223 (42.3)	226 (45.5)	<0.001
HDL_C, mean (SD), mg/dL	58.0 (14.9)	51.6 (13.3)	<0.001
TyG, mean (SD)	8.7 (0.6)	9.0 (0.5)	<0.001
TyG-BMI, mean (SD)	232 (44.9)	273 (49.9)	<0.001
TyG-WC, mean (SD)	768 (138)	897 (133)	<0.001
TyG-WHTR, mean (SD)	4.6 (0.8)	5.3 (0.8)	<0.001
TG/HDL_C ratio, mean (SD)	2.9 (2.3)	4.4 (3.1)	<0.001
METS-IR, mean (SD)	38.6 (8.4)	46.2 (9.5)	<0.001
CMI, mean (SD)	0.7 (0.6)	1.1 (0.8)	<0.001

^a^In UK Biobank, household income <£18,000 was defined as low household income, High income level referred to household income >£52000.

—, data not available.

CVD, cardiovascular disease; SD, standard deviation; BMI, body mass index; WC, waist circumference; HDL_C, high density lipoprotein_ cholesterol, TC, total cholesterol; TG, triglyceride; FBG, Fasting blood glucose; IR, insulin resistance.

### 3.2 Associations of seven IR-related indices with adverse cardiovascular events

**Figure 1:**
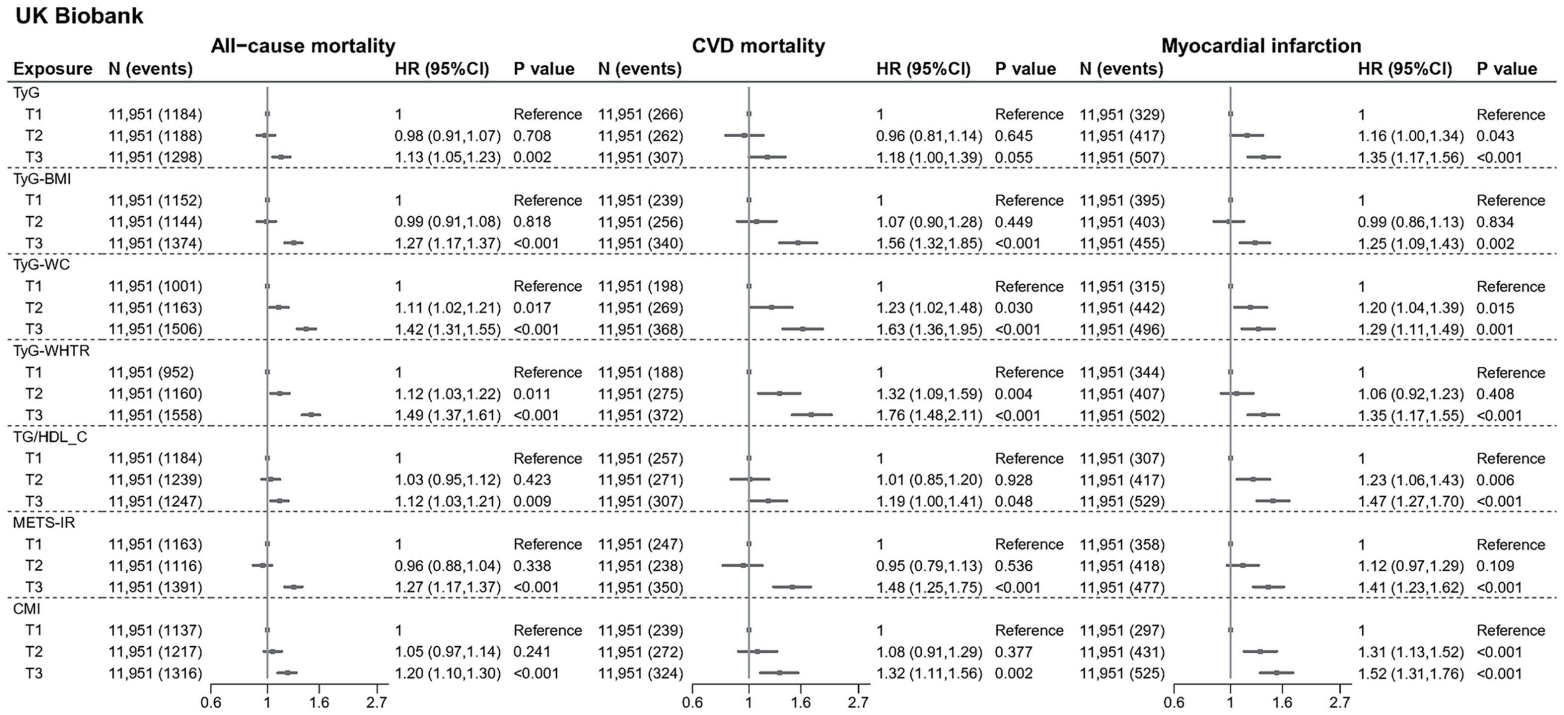
Associations of insulin resistance (IR)-related indices with adverse cardiovascular events. UKBB: Model was adjusted by age (continuous), sex (male, female), ethnicity/race (White, Asian or Asian British, Black or Black British, Chinese, Mixed, Other ethnic group), educational level (less than high school, high school and above), smoking status (Yes or No), alcohol consumption (continuous), physical activity (adequate, inadequate), TC (continuous), Townsend deprivation index (three categories stratified based on tertiles). *p*-values less than 0.05 (*p* < 0.05) were considered significant. IR, insulin resistance; TyG, triglyceride-glucose; BMI, body mass index; WC, waist circumference; WHTR, waist circumference/height ratio; TG, triglyceride; HDL-C, High density lipoprotein-cholesterol; METSIR, Metabolic SCORE for insulin resistance; CMI, cardiometabolic index; CVD, cardiovascular disease; HR, hazard ratio; CI, confidence interval; N, number.

As shown in Figure 1, all seven indices demonstrated graded cardiovascular risk stratification when analyzed by tertiles. In the UKBB cohort (Model 2), all metrics except TyG index exhibited significantly elevated risks for all-cause mortality, CVD mortality, and myocardial infarction in the highest tertile (T3 vs. T1: HR range: all-cause mortality: 1.12–1.49; CVD mortality: 1.18–1.76; myocardial infarction: 1.25–1.52). Model 2 observations remained consistent across Model 1 analyses in UKBB (Supplementary Table S3).

Subgroup analyses of HUA patients with comorbid diabetes revealed concordant patterns with overall cohort (Supplementary Table S4). Notably, only selected indices maintain significance for CVD mortality: TyG-BMI [HR:1.34, 95%CI:(1.06–1.71)], TyG-WC [1.43 (1.11–1.85)], TyG-WHTR [1.48 (1.16–1.90)], and METS-IR [1.47 (1.15–1.88)] in UKBB. Non-HUA individuals exhibited differential associations. The UKBB cohort demonstrated robust correlations between all seven indices and cardiovascular endpoints, particularly myocardial infarction (HRs > 1.70 across metrics) (Supplementary Table S5).

Furthermore, we observed a higher risk of all-cause mortality associated with higher levels of TyG, METS-IR and TyG-WHTR compared to lower levels in the Shanghai Pudong cohort [TyG: HR:1.16, 95%CI:(1.00–1.34); TyG-WHTR: 1.16(1.03–1.30); METS-IR: 1.15(1.02–1.29)], (Table 2).

**Table 2:** The TyG, TyG WC, TyG BMI, TyG WHTR, TG/HDL_C, METS-IR and CMI association with all-cause mortality calculated using cox proportional hazards model in the Shanghai Pudong cohort.

Case/N		HR (95%CI)	*p*-value	Case/N	HR (95%CI)	*p*-value	Case/N	HR (95%CI)	*p*-value	Case/N	HR (95%CI)	*p*-value
TyG				TyG-BMI			TyG-WC			TyG-WHTR		
Model 1												
T1	388/4227	1	Ref	421/4227	1	Ref	336/4227	1	Ref	329/4227	1	Ref
T2	381/4228	1.04(0.90,1.20)	0.573	331/4226	0.84(0.73,0.97)	0.020	362/4226	0.95(0.82,1.10)	0.484	336/4226	0.92(0.79,1.07)	0.278
T3	393/4225	1.13(0.99,1.31)	0.080	410/4227	1.04(0.91,1.20)	0.545	464/4227	1.13(0.98,1.30)	0.094	497/4227	1.13(0.98,1.30)	0.090
Model 2												
T1	388/4227	1	Ref	421/4227	1	Ref	336/4227	1	Ref	329/4227	1	Ref
T2	381/4228	1.11(0.96,1.29)	0.143	331/4226	1.08(0.93,1.25)	0.310	362/4226	1.00(0.86,1.16)	0.977	336/4226	1.02(0.90,1.15)	0.798
T3	393/4225	1.16(1.00,1.34)	0.049	410/4227	1.14(0.99,1.31)	0.062	464/4227	1.11(0.96,1.28)	0.147	497/4227	1.16(1.03,1.30)	0.013

TG/HDL_C				METS-IR			CMI		
Model 1									
T1	383/4227	1	Ref	385/4227	1	Ref	372/4227	1	Ref
T2	368/4226	0.96(0.83,1.10)	0.545	336/4226	0.86(0.74,1.00)	0.047	374/4226	0.94(0.82,1.09)	0.429
T3	411/4227	1.05(0.91,1.21)	0.486	441/4227	1.07(0.93,1.22)	0.363	416/4227	1.03(0.89,1.18)	0.701
Model 2									
T1	382/4254	1	Ref	384/4251	1	Ref	372/4251	1	Ref
T2	369/4248	1.04(0.92,1.17)	0.560	338/4251	1.10(0.97,1.25)	0.136	374/4251	1.00(0.89,1.13)	0.976
T3	412/4251	1.08(0.96,1.22)	0.207	441/4251	1.15(1.02,1.29)	0.021	417/4251	1.08(0.96,1.22)	0.210

Model was adjusted by age (continuous), sex (male, female), educational level (less than high school, high school and above), smoking status (Yes or No), drinking status (Yes or No), physical activity (adequate, inadequate), TC (continuous).

BMI, body mass index; WC, waist circumference; HDL_C, High density lipoprotein_ cholesterol; TC, total cholesterol; TG, triglyceride; FBG, Fasting blood glucose; IR, insulin resistance.

### 3.3 Associations of continuous seven IR-related indices, SCORE2 and PREVENT equations with adverse cardiovascular events

**Figure 2:**
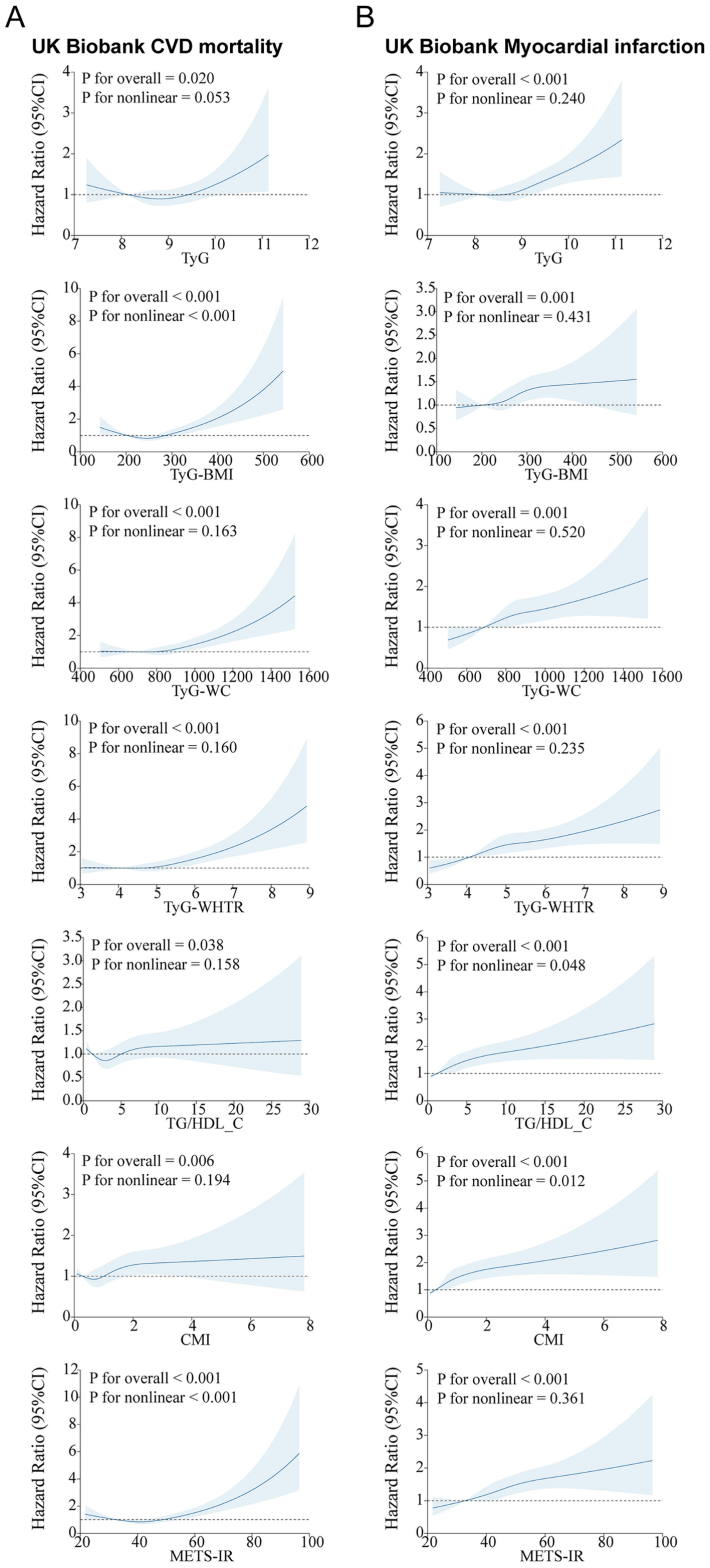
Restricted cubic spline (RCS) curve illustrating the associations of insulin resistance (IR)-related indices with adverse cardiovascular events. UKBB: Model was adjusted by age (continuous), sex (male, female), ethnicity/race (White, Asian or Asian British, Black or Black British, Chinese, Mixed, Other ethnic group), educational level (less than high school, high school and above), smoking status (Yes or No), alcohol consumption (continuous), physical activity (adequate, inadequate), TC (continuous), Townsend deprivation index (three categories stratified based on tertiles). RCS, Restricted cubic spline; IR, insulin resistance; TyG, triglyceride-glucose; BMI, body mass index; WC, waist circumference; WHTR, waist circumference/height ratio; TG, triglyceride; HDL-C, High density lipoprotein-cholesterol; METSIR, Metabolic SCORE for insulin resistance; CMI, cardiometabolic index; CVD, cardiovascular disease. **(A)** IR-related indices to CVD morality. **(B)** IR-related indices to Myocardial infarction.

Nonlinear associations between the seven indices and adverse cardiovascular events were observed (Figure 2, Supplementary Figure S3). In UKBB, TyG-BMI and METS-IR exhibited significant hook-shaped relationships with CVD mortality, whereas the TG/HDL-C ratio and CMI showed a consistently increasing trend in myocardial infarction risk. In addition, SCORE2 demonstrated nonlinear relationships with both CVD mortality and myocardial infarction, while the PREVENT equation showed a nonlinear association with CVD mortality (Supplementary Figure S4).

### 3.4 Subgroup analyses by sex and age

Subgroup analyses accounting for sex and age effects is presented in Supplementary Tables S6, S7. The UKBB cohort demonstrated significant multiplicative interaction between TG/HDL-C ratio and sex for CVD mortality (*p*-value, 0.022). Female participants in the highest tertile (T3) of TyG-WC, TG/HDL-C, METS-IR, and CMI exhibited elevated all-cause mortality risk compared to male counterparts in T1 (HR range:1.03–1.30). Among elderly participants (≥65 years), TyG-WHTR and METS-IR at T2 significantly increased CVD mortality risk versus T1 [HR: 4.53,95%CI: (3.54–5.80) and 3.31 (2.59–4.23), respectively].

### 3.5 Mediating effects of biochemical indicators and accelerated aging

**Figure 3:**
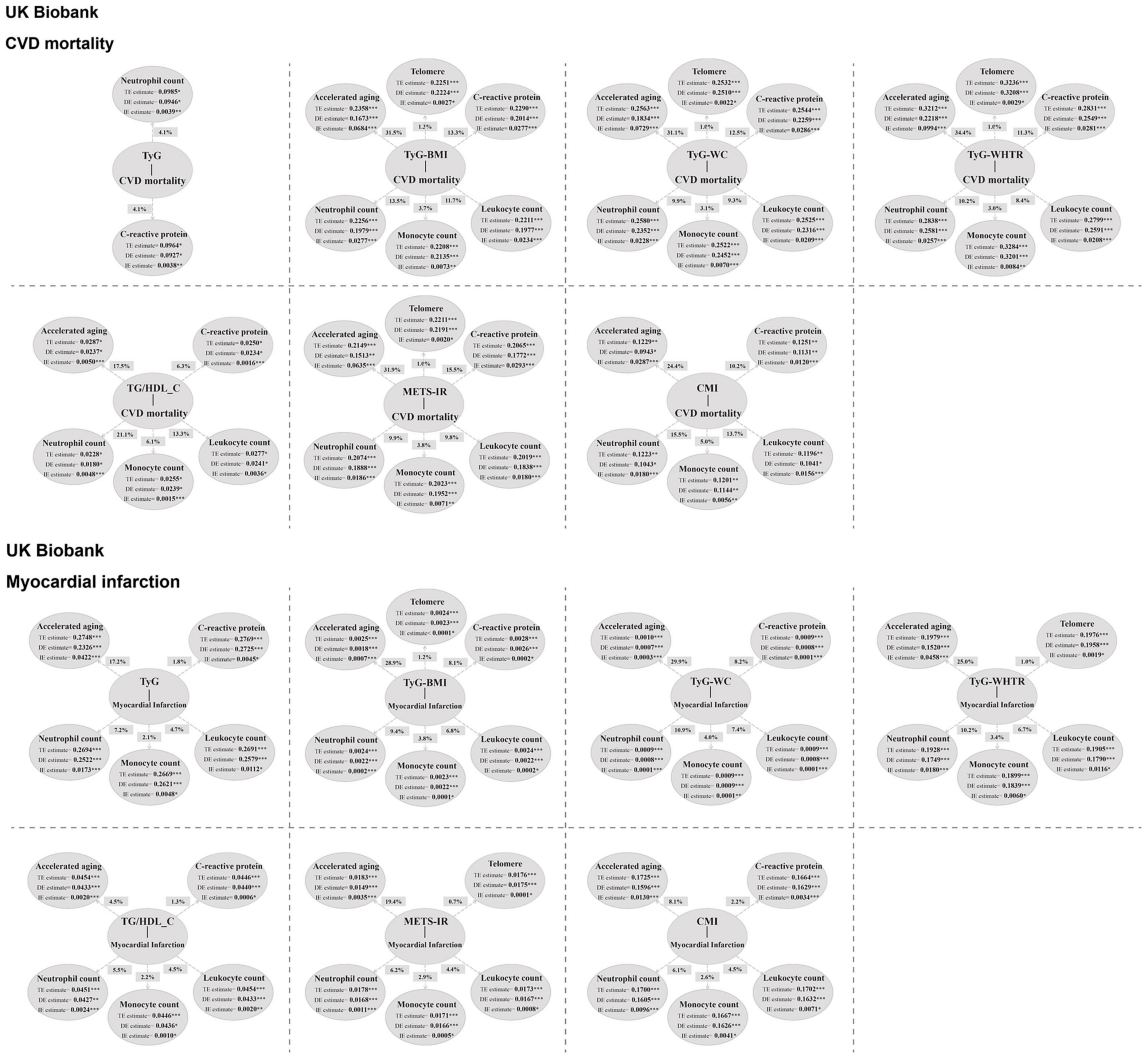
Inflammatory markers and phenotypic aging mediate the associations of insulin resistance (IR)-related indices with adverse cardiovascular events in UKBB. Model was adjusted by age (continuous), sex (male, female), ethnicity/race (White, Asian or Asian British, Black or Black British, Chinese, Mixed, Other ethnic group), educational level (less than high school, high school and above), smoking status (Yes or No), alcohol consumption (continuous), physical activity (adequate, inadequate), TC (continuous), Townsend deprivation index (three categories stratified based on tertiles). IR, insulin resistance; UKBB, UK Biobank; TyG, triglyceride-glucose; BMI, body mass index; WC, waist circumference; WHTR, waist circumference/height ratio; TG, triglyceride; HDL-C, High density lipoprotein-cholesterol; METSIR, Metabolic SCORE for insulin resistance; CMI, cardiometabolic index; CVD, cardiovascular disease.

Mediation analyses were conducted in both cohorts to explore the roles of inflammatory markers and aging acceleration (Figure 3, Supplementary Figure S5). Within the UKBB cohort, neutrophil count and CRP exerted positive mediating effects in the associations between the seven indices and CVD mortality. Specifically, mediation proportions for neutrophil count vs. CRP were as follows: TyG (4.1% vs. 4.1%), TyG-BMI (13.5% vs. 13.3%), TyG-WC (9.9% vs. 12.5%), TyG-WHTR (10.2% vs. 11.3%), TG/HDL-C ratio (21.1% vs. 6.3%), METS-IR (9.9% vs. 15.5%), and CMI (15.5% vs. 10.2%). Leukocyte and monocyte count mediated effects for six exposures excluding TyG, with mediation ranges of 8.4–13.7% (leukocytes) and 3.0–6.1% (monocytes). Leukocyte telomere length had weaker mediation in three indices: TyG-BMI (1.3%), TyG-WC (1.0%), and TyG-WHTR (1.0%). For myocardial infarction, four mediators (leukocyte count, monocyte count, neutrophil count, and CRP) demonstrated notable mediation across all seven exposures, with proportions ranging from 1 to 11%. Phenotypic age acceleration emerged as the predominant mediator (17.5–34.4%), exceeding inflammatory pathway contributions.

### 3.6 Sensitivity analyses

To assess the robustness of UKBB cohort findings, sensitivity analyses were performed by stratifying the HUA, non-HUA, and HUA with diabetes subgroups into quartiles and repeating Cox proportional hazards modeling. In the HUA population of the UKBB cohort, TyG-WHTR significantly increased the risk for all outcomes in both models (Q4 vs. Q1: HR range: 1.28–1.95) (Supplementary Table S8). For individuals without HUA, seven metrics significantly increased the risk of myocardial infarction (Q4 vs. Q1: HR range: 1.81–2.40) (Supplementary Table S9). In the HUA-combined diabetic population, higher TyG-WHTR was significantly associated with increased CVD mortality [Q4 vs. Q1: HR: 2.59, 95%CI: (1.56–4.31)] (Supplementary Table S10). After additional adjustment for major clinical comorbidities and medications, the associations between IR-related indices and cardiovascular outcomes remained robust, with TyG-WHTR T3 still exhibiting the highest risk for CVD mortality [HR: 1.76, 95% CI: (1.48–2.11), Supplementary Table S11]. In the competing risk analysis, the associations remained robust, with TyG-WHTR showing a significant association with CVD mortality [sHR: 1.365, 95% CI: (1.245–1.497), *p* < 0.001], consistent with the primary Cox model findings (Supplementary Table S12).

### 3.7 ROC curves of time-dependent and random forest

**Figure 4:**
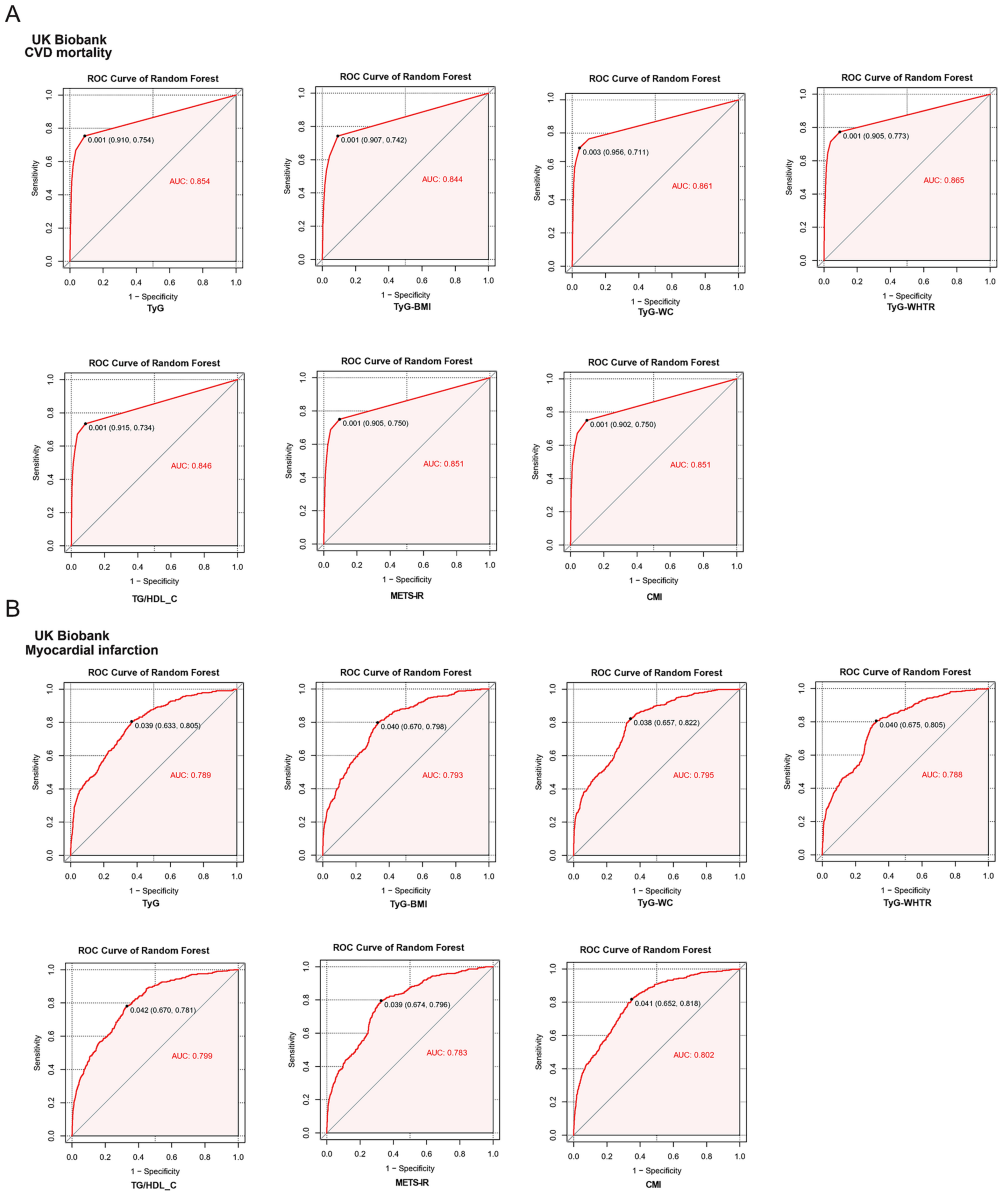
Time-dependent ROC curves and time-dependent AUC values of the insulin resistance (IR)-related indices, SCORE2 and PREVENT for predicting 10 years-adverse cardiovascular events. UKBB: Model was adjusted by age (continuous), sex (male, female), ethnicity/race (White, Asian or Asian British, Black or Black British, Chinese, Mixed, Other ethnic group), educational level (less than high school, high school and above), smoking status (Yes or No), alcohol consumption (continuous), physical activity (adequate, inadequate), TC (continuous), Townsend deprivation index (three categories stratified based on tertiles). ROC, Receiver operating characteristic; AUC, Area under the curve; TyG, triglyceride-glucose; BMI, body mass index; WC, waist circumference; WHTR, waist circumference/height ratio; TG, triglyceride; HDL-C, High density lipoprotein-cholesterol; METSIR, Metabolic score for insulin resistance; CMI, cardiometabolic index; CVD, cardiovascular disease. **(A)** IR-related indices to CVD morality. **(B)** IR-related indices to Myocardial infarction.

Based on the time-dependent ROC analysis, TyG-WHTR showed predictive performance comparable to SCORE2 for 10-year CVD mortality in UKBB (TyG-WHTR: AUC, 0.7328, SCORE2: AUC, 0.7316) (Figure 4). SCORE2 demonstrated the best performance in predicting ten-year CVD risk related to UKBB (AUC: myocardial infarction: 0.6808, stroke: 0.6828, ischemic stroke: 0.6934). In the random forest analysis based on a 70% training set and an independent 30% test set, the AUC values for predicting binary cardiovascular outcomes ranged from 0.798 to 0.930. Similar model performance was observed in the additional 5-fold cross-validation analysis, supporting the robustness of the findings (Supplementary Figures S6, S7). In the UKBB, TyG-WHTR exhibited better CVD mortality prediction than SCORE2 (AUC: 0.862 VS 0.846), while SCORE 2 demonstrated best performance in prediction of ischemic stroke (AUC, 0.833).

### 3.8 Genetic associations with cardiovascular risk factors and adverse cardiovascular events

**Figure 5:**
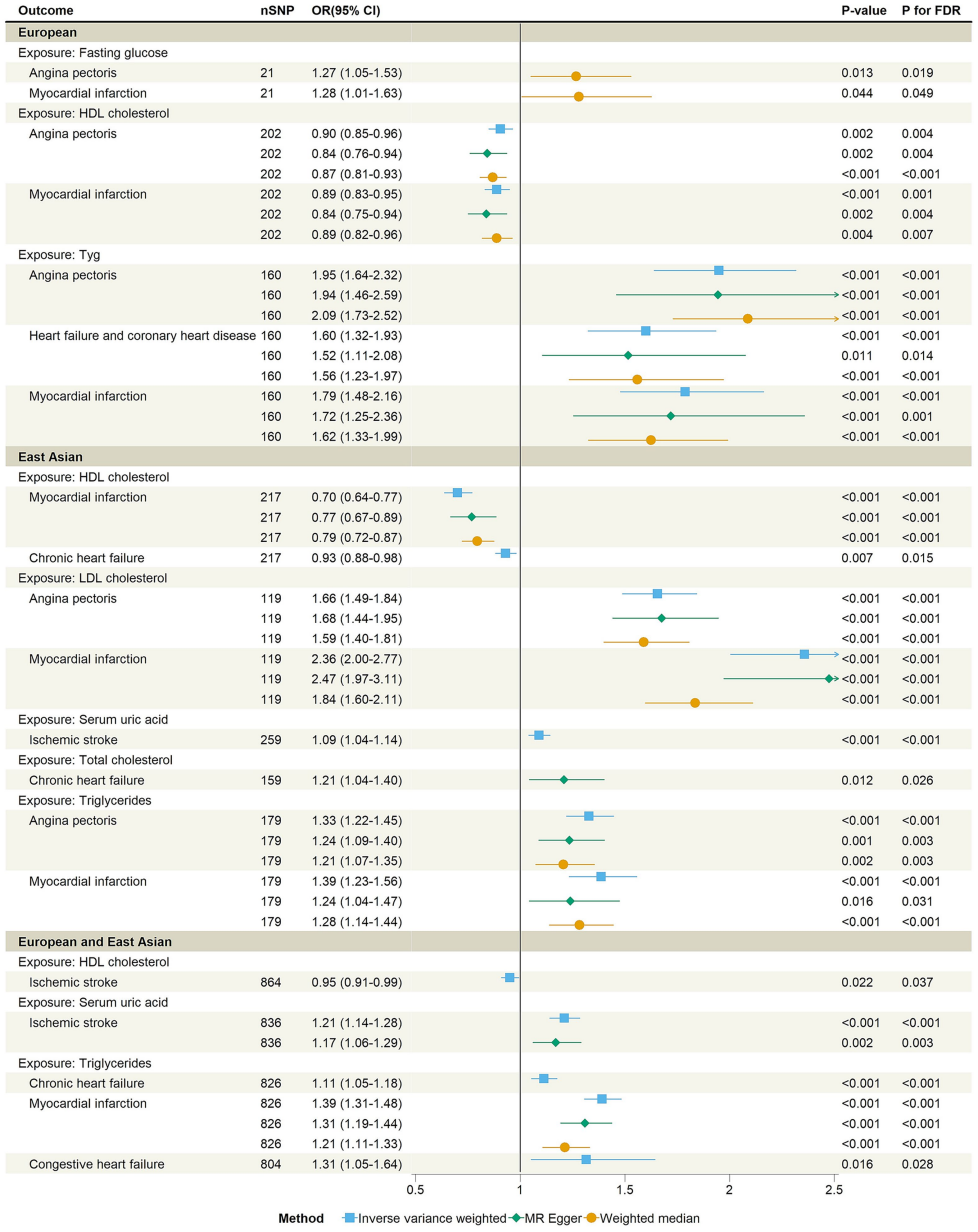
Genetically predicted insulin resistance (IR)-related indices associated with adverse cardiovascular events. *p*-values less than 0.05 (*p* < 0.05) were considered significant. IR, insulin resistance; TyG, triglyceride-glucose; HDL, high density lipoprotein; LDL, low density lipoprotein; nSNP, non-synonymous single nucleotide polymorphism; OR, odds ratio; CI, confidence interval; FDR, false discovery rate.

IVW analyses employing random-effects models suggested potential causal associations between genetically determined lipid profiles and cardiovascular outcomes (Figure 5). In European populations, genetically predicted elevated fasting blood glucose was significantly associated with higher risks of angina pectoris [OR: 1.27, 95%CI: (1.05–1.53)] and myocardial infarction [1.28 (1.01–1.63)]. Genetically elevated HDL cholesterol demonstrated protective effects against angina pectoris [0.90 (0.85–0.96)] and myocardial infarction [0.89 (0.83–0.95)]. Conversely, a genetically predicted TyG index increase was associated with heightened risks of angina pectoris [1.95 (1.64–2.32)], heart failure/coronary artery disease [1.60 (1.32–1.93)], and myocardial infarction [1.79 (1.48–2.16)]. East Asian analyses showed HDL cholesterol inversely associated with myocardial infarction [0.70 (0.64–0.77)] and chronic heart failure [0.93 (0.88–0.98)]. LDL cholesterol exhibited positive causal relationships with angina pectoris [1.66 (1.49–1.84)] and myocardial infarction [2.36 (2.00–2.77)], while triglycerides demonstrated similar patterns for angina [1.33 (1.22–1.45)] and myocardial infarction [1.39 (1.23–1.56)]. Serum uric acid showed consistent ischemic stroke risk elevation across ethnicities [Europeans:1.09 (1.04–1.14); Mixed:1.21 (1.14–1.28)]. In multi-ethnic analyses, HDL cholesterol retained inverse associations with ischemic stroke [0.95 (0.91–0.99)]. Triglycerides were positively associated with chronic heart failure [1.11 (1.05–1.18)], myocardial infarction [1.39 (1.31–1.48)], and congestive heart failure [1.31 (1.05–1.64)]. Sensitivity analyses using MR-PRESSO and Steiger directionality tests confirmed the robustness of the genetic findings, showing no significant outlier SNPs and strongly supporting the hypothesized causal direction from TyG index to cardiovascular outcomes (Supplementary Tables S13, S14).

## 4 Discussion

This study comprehensively evaluated seven IR-related indices and adverse cardiovascular outcomes in HUA populations using two large prospective cohorts from the UK and the China. Our analyses demonstrated relationships between elevated IR biomarkers and cardiovascular risk escalation among HUA patients. TyG-WHTR emerged as the optimal predictor for CVD mortality, while CMI showed superior discriminatory capacity for myocardial infarction. Mechanistically, CRP and leukocyte telomere length partially mediated these associations. Sensitivity analyses incorporating multiple adjustment models and subgroup stratifications confirmed outcome stability across demographic and clinical subgroups.

Our findings confirm the robust cardiovascular risk predictive capacity of IR-related indices in HUA, consistent with existing evidence while expanding clinical insights. Established multifactorial algorithms, such as the SCORE2 model (developed by the ESC for European populations to estimate 10-year ASCVD risk) and the PREVENT equations (calibrated by the AHA for multi-ethnic US cohorts), demonstrated reliable predictive efficacy for 10-year myocardial infarction and ischemic stroke within the UKBB. TyG-WHTR demonstrated robust predictive utility for 10-year CVD mortality, with predictive performance comparable to SCORE2, while also being a simple and easily obtainable insulin resistance-related index. This aligns with previous U.S. findings reporting a 66% increased risk (20), suggesting that while general-population scores are robust, IR indices may offer superior sensitivity for mortality within the specific metabolic milieu of HUA. While the PREVENT equation effectively stratifies long-term mortality risk, random forest modeling revealed that TyG-WHTR significantly enhances the precision of individualized risk identification. Similarly, the CMI showed the strongest association with myocardial infarction (HR, 1.52), consistent with population-based studies demonstrating 41% increased risk (32). Compared to the Mashhad cohort study (TyG and TG/HDL-C ratio HRs of 1.63 and 1.07) (33), our analysis observed more pronounced HUA-related risk elevations (1.66 and 1.67). This underscores that HUA synergizes with IR to amplify vascular damage, likely because uric acid impairs insulin signaling, induces myocardial IR, and reduces glucose uptake (10, 34). Although multifactorial tools are clinical gold standards, they were calibrated for broader reference populations and specific primary outcomes. Our subgroup findings highlight the clinical targeting value of IR-related indices. The stronger associations observed in elderly individuals and women suggest that TyG-WHTR and similar markers could serve as high-priority early screening tools for these vulnerable groups within the hyperuricemia population. Simple IR-related indices such as TyG-WHtR and CMI may provide an accessible and cost-effective “first-line flag” for identifying HUA patients who may require further cardiovascular risk assessment, although they should be considered complementary to, rather than replacements for, established risk scores such as SCORE2 and PREVENT.

Our investigation revealed threshold-dependent associations between specific IR markers and cardiovascular outcomes, paralleling previous epidemiological evidence demonstrating nonlinear J-curve relationships of the TyG index with all-cause mortality (threshold 9.36) and cardiovascular mortality (threshold 9.52) (35). Notably, MR analysis substantiated genetic causality between TyG and CVD, demonstrating that genetically elevated TyG significantly increased cardiovascular susceptibility in European populations. Specifically, each standard deviation increases in genetically predicted TyG corresponded to elevated risks of angina pectoris (OR, 1.95), myocardial infarction (OR, 1.79), and composite coronary events including heart failure and coronary artery disease (OR, 1.60), reinforcing conclusions from prospective cohort investigations (36). Weak-instrument bias, residual horizontal pleiotropy, sample overlap between exposure and outcome datasets, and population mismatch may influence the MR estimates. Therefore, the MR results should be interpreted as supportive evidence rather than definitive confirmation of causality.

Our analysis substantiates the complex reciprocal pathophysiology between IR and biological aging. Mechanistic studies reveal that IR accelerates cellular senescence processes in critical tissues including neurons and adipose depots, whereas age-related declines in insulin sensitivity and metabolic clearance reciprocally exacerbate IR progression (37, 38). Epidemiological evidence confirms an inflammation-amplified, independent association between IR severity and validated aging biomarkers, synergistically elevating cardiovascular risk profiles (39). The hyperinsulinemic state perpetuates chronic inflammation through NF-κB pathway activation, driving systemic elevation of proinflammatory mediators including IL-6 and TNF-*α* (40). Concomitant impairment of insulin clearance capacity induces mitochondrial oxidative stress, telomeric erosion, and endothelial senescence initiation. These processes culminate in senescence-associated secretory phenotype (SASP) propagation that promotes monocyte-mediated lipid accumulation and atherosclerotic foam cell formation (41, 42). Our cohort analysis demonstrated significant positive associations between validated aging metrics (phenotypic age acceleration and leukocyte telomere attrition) with incident cardiovascular events and mortality, establishing biological aging as a critical mediator of cardiovascular pathogenesis. Through comprehensive mediation analysis in the UK Biobank population, we identified that neutrophil-mediated inflammation accounted for 21.1% of the TG/HDL-C-associated CVD mortality risk, and phenotypic age acceleration contributes to over 40% of IR-related cardiovascular risk. These findings highlight a potential role of accelerated aging in linking metabolic dysregulation to adverse clinical outcomes. These estimates require cautious interpretation as the observational design restricts definitive causal mediation inference and unmeasured confounding from diet and medication use may bias the observed proportions. Our results support plausible mechanistic associations rather than conclusive biological causality.

This study has several strengths. Incorporation of a multinational cohort exceeding 36,000 participants substantially improves statistical power and generalizability. Comprehensive evaluation of seven validated IR-related indices addresses a critical knowledge gap in understanding cardiometabolic risk among HUA populations. Pioneering exploration of inflammation and accelerated aging as mechanistic mediators between IR and cardiovascular sequelae. We compared the predictive performance of IR-related indices, SCORE2 and the PREVENT equation in CVD incidence and mortality in the UKBB. Although TyG-WHTR showed comparable discriminatory power to SCORE2, we did not evaluate its clinical net benefit using NRI/IDI or decision-curve analysis. Several methodological considerations merit discussion. The exclusion criteria for missing covariates or incomplete follow-up, while ensuring analytical robustness, may introduce survival bias and limit population representativeness. Cardiovascular outcome ascertainment in Pudong cohort participants relied on self-reported diagnoses susceptible to under detection and recall inaccuracies inherent to retrospective designs. The absence of serial biomarker measurements precludes assessment of temporal relationships between dynamic IR fluctuations and disease progression. Another limitation is the inability to distinguish between asymptomatic hyperuricemia and symptomatic gout due to data constraints. Given that gout signifies a higher inflammatory burden and greater disease severity, the inherent heterogeneity within the HUA cohort might result in a combined risk estimation for individuals across different stages of the disease spectrum. Furthermore, the absence of data on urate-lowering therapies, such as allopurinol, represents a notable limitation. This could lead to the misclassification of treated individuals with normalized serum uric acid levels into the normouricemic group, potentially resulting in a conservative underestimation of the actual association between hyperuricemia and adverse cardiovascular outcomes. Although we used phenotypic age acceleration, calculated as the residual of phenotypic age regressed on chronological age, rather than raw phenotypic age, this residualization cannot fully remove the influence of shared biomarker components. Finally, although we rigorously adjusted for major confounders, residual confounding from unmeasured variables (e.g., subclinical inflammation) persists, necessitating confirmation through prospective studies incorporating multimodal omics profiling and digital health monitoring technologies. In MR analysis, the TyG index, a composite indicator of triglycerides and blood glucose, cannot completely eliminate horizontal pleiotropy at its inherent level. Insufficient sample sizes in some GWAS led to relaxed thresholds. Although weak instrument bias was excluded via F-statistics, larger samples are still needed for validation. There is potential sample overlap among East Asian and mixed populations. Despite robust results verified by multiple quality controls, replication validation using independent cohorts remains necessary.

## 5 Conclusion

In conclusion, we demonstrated robust associations between all evaluated IR metrics and adverse cardiovascular events in HUA, with the TyG-WHTR demonstrated robust predictive utility for CVD mortality. Mechanistic analyses identified inflammatory biomarkers and phenotypic age acceleration as principal mediators of IR-related cardiovascular pathogenesis. These findings underscore the need for future investigations to optimize cardiovascular risk stratification in hyperuricemia populations. While SCORE2 and PREVENT equations demonstrate efficacy in stratifying long-term cardiovascular events and mortality, incorporating insulin resistance indices enhances the precision of personalized preventive strategies. In future, we will implement systematic cardiovascular prognosis monitoring protocols, and advance molecular-level characterization of inflammatory cascades and phenotypic remodeling processes that underlie IR-induced cardiovascular deterioration.

## Data Availability

The raw data supporting the conclusions of this article will be made available by the authors, without undue reservation.

## Footnotes

1
http://links.lww.com/JS9/C792

2
https://gwas.mrcieu.ac.uk/

3
https://r12.finngen.fi/
