# Supplementary material for: Comparative evaluation of insulin resistance indices for predicting adverse cardiovascular events in hyperuricemia: insights from UK biobank and Shanghai Pudong cohort
Source: Front Nutr. 2026 Jul 13;13:1870901. doi: 10.3389/fnut.2026.1870901 (PMC13402903; doi:10.3389/fnut.2026.1870901)
Supplement: Supplementary file 1 [file Supplementary_file_1.DOCX]

SUPPLEMENTAL MATERIAL

**Table S1.** SCORE2 risk prediction algorithms1.

Variables used Calculation formula

Age

Total cholesterol (mmol/l)

HDL cholesterol SBP (mmHg) Diabetes Smoking

∑β (x − xcen) = Log SHR of age × (mean of age - 60)/5

+ Log SHR of total cholesterol × (mean of total cholesterol - 6)

+ Log SHR of HDL cholesterol × (mean of HDL cholesterol- 1.3) /0.5

+ Log SHR of SBP × (mean of SBP - 120)/20

+ Log SHR of diabetes × mean of diabetes

+ Log SHR of smoking × mean of smoking

+ Log SHR of total cholesterol interaction with age × (mean of age - 60)/5 × (mean of total cholesterol - 6)

+ Log SHR of HDL interaction with age × (mean of age - 60)/5 × (mean of HDL cholesterol- 1.3) /0.5

+ Log SHR of SBP interaction with age × (mean of age - 60)/5 × (mean of SBP - 120)/20

+ Log SHR of diabetes interaction with age × (mean of age - 60)/5 × mean of diabetes

+ Log SHR of smoking interaction with age × (mean of age - 60)/5 × mean of smoking 10-year risk = 1 - 0.9605^exp(∑ β (x − xcen))

**Note:** SHR=subdistribution hazard ratios.

**Reference:**

1. SCORE2 risk prediction algorithms: new models to estimate 10-year risk of cardiovascular disease in Europe. Eur Heart J [Internet]. 2021 Jan 7 [cited 2025 May

18];42(25). Available from: <http://pubmed-ncbi-nlm-nih-gov-s.webvpn.njmu.edu.cn:8118/34120177/>

## **Table S2.** The American Heart Association’s PREVENT Equations1.

Variables used Calculation formula

Age

Total cholesterol HDL cholesterol SBP

Diabetes Smoking eGFR

Anti-hypertensive medication use Statin use

**log-Odds (Female)** = -3.307728 + 0.7939329 × (age – 55) /10 + 0.0305239 × (Tc – HDL – 3.5) – 0.1606857 × (HDL – 1.3) /0.3 – 0.2394003 × (min(SBP, 110) – 110) /20 + 0.360078 × (ma×(SBP, 110) –

130) /20 + 0.8667604 × (if diabetes) + 0.5360739 × (if current smoker) + 0.6045917 × (min(eGFR, 60) –

60) / -15 + 0.0433769 × (ma×(eGFR, 60) – 90) / -15 + 0.3151672 × (if using anti-hypertensive medication) – 0.1477655 × (if using statin) – 0.0663612 × (if using anti-hypertensive medication) × (ma×(SBP, 110) –

130) /20 + 0.1197879 × (if using statin) × (Tc – HDL – 3.5) – 0.0819715 × (age – 55) /10 × (Tc – HDL –

3.5) + 0.0306769 × (age – 55) /10 × (HDL – 1.3) /0.3 – 0.0946348 × (age – 55) /10 × (ma×(SBP, 110) –

130) /20 – 0.27057 × (age – 55) /10 × (if diabetes) – 0.078715 × (age – 55) /10 × (if current smoker) – 0.1637806 × (age – 55) /10 × (min(eGFR, 60) – 60) / -15

**log-Odds (Male)** = -3.031168 + 0.7688528 × (age – 55) /10 + 0.0736174 × (Tc – HDL – 3.5) –

0.0954431 × (HDL – 1.3) /0.3 – 0.4347345 × (min(SBP, 110) – 110) /20 + 0.3362658 × (ma×(SBP, 110) –

130) /20 + 0.7692857 × (if diabetes) + 0.4386871 × (if current smoker) + 0.5378979 × (min(eGFR, 60) –

60) / -15 + 0.0164827 × (ma×(eGFR, 60) – 90) / -15 + 0.288879 × (if using anti-hypertensive medication) – 0.1337349 × (if using statin) – 0.0475924 × (if using anti-hypertensive medication) × (ma×(SBP, 110) –

130) /20 + 0.150273 × (if using statin) × (Tc – HDL – 3.5) – 0.0517874 × (age – 55) /10 × (Tc – HDL –

3.5) + 0.0191169 × (age – 55) /10 × (HDL – 1.3) /0.3 – 0.1049477 × (age – 55) /10 × (ma×(SBP, 110) –

130) /20 – 0.2251948 × (age – 55) /10 × (if diabetes) – 0.0895067 × (age – 55) /10 × (if current smoker) –

0.1543702 × (age – 55) /10 × (min(eGFR, 60) – 60) / -15

**Risk** = exp(log-Odds) / (1 + exp(log-Odds))

**Note:** eGFR=estimated glomerular filtration rate.

**Reference:**

1. Khan Ss, Matsushita K, Sang Y, Ballew Sh, Grams Me, Surapaneni A, et al. Development and Validation of the American Heart Association’s PREVENT Equations. Circulation [Internet]. 2024 Jun 2 [cited 2024 Aug 3];149(6). Available from: <http://pubmed-ncbi-nlm-nih-gov-s.webvpn.njmu.edu.cn:8118/37947085/>

**Table S3.** Associations of seven insulin resistance-related indices with adverse cardiovascular events calculated using Cox proportional hazards model in the patients with hyperuricemia in UK Biobank.

**UK Biobank Variable Range CVD mortality Myocardial infarction Stroke Ischemic stroke All-cause mortality**

| **Model 1**  **TyG** |  | **HR (95%CI)** | **P-value** | **HR (95%CI)** | **P-value** | **HR (95%CI)** | **P-value** | **HR (95%CI)** | **P-value** | **HR (95%CI)** | **P-value** |
| --- | --- | --- | --- | --- | --- | --- | --- | --- | --- | --- | --- |
| T1 | 6.8-8.8 | 1 | ref | 1 | ref | 1 | ref | 1 | ref | 1 | ref |
| T2 | 8.8-9.2 | 0.94(0.80,1.12) | 0.515 | 1.23(1.06,1.42) | 0.005 | 1.06(0.92,1.23) | 0.395 | 1.13(0.96,1.31) | 0.135 | 0.97(0.89,1.05) | 0.404 |
| T3 | 9.2-11.5 | 1.16(0.98,1.37) | 0.078 | 1.49(1.30,1.71) | <0.001 | 0.99(0.86,1.15) | 0.937 | 1.07(0.91,1.25) | 0.423 | 1.11(1.03,1.20) | 0.008 |
| **TyG-BMI**  T1 | 129.3-248.6 | 1 | ref | 1 | ref | 1 | ref | 1 | ref | 1 | ref |
| T2 | 248.6-287.4 | 1.11(0.93,1.32) | 0.264 | 1.02(0.89,1.17) | 0.761 | 1.04(0.90,1.20) | 0.611 | 1.03(0.88,1.20) | 0.713 | 1.02(0.94,1.10) | 0.701 |
| T3 | 287.4-715.9 | 1.71(1.44,2.01) | <0.001 | 1.30(1.13,1.49) | <0.001 | 1.05(0.91,1.21) | 0.502 | 1.08(0.93,1.27) | 0.316 | 1.35(1.25,1.47) | <0.001 |
| **TyG-WC**  T1 | 476.9-837.6 | 1 | ref | 1 | ref | 1 | ref | 1 | ref | 1 | ref |
| T2 | 837.6-946.5 | 1.26(1.05,1.52) | 0.014 | 1.24(1.07,1.44) | 0.004 | 1.01(0.87,1.17) | 0.935 | 1.02(0.87,1.20) | 0.806 | 1.13(1.04,1.24) | 0.004 |
| T3 | 946.5-1875.5 | 1.79(1.49,2.13) | <0.001 | 1.36(1.18,1.58) | <0.001 | 1.16(1.01,1.35) | 0.043 | 1.20(1.02,1.41) | 0.024 | 1.54(1.41,1.67) | <0.001 |
| **TyG-WHTR**  T1 | 2.8-4.9 | 1 | ref | 1 | ref | 1 | ref | 1 | ref | 1 | ref |

| T2 | 4.9-5.5 | 1.35(1.12,1.63) | 0.001 | 1.11(0.96,1.28) | 0.173 | 1.12(0.97,1.30) | 0.135 | 1.10(0.94,1.29) | 0.251 | 1.14(1.05,1.25) | 0.002 |
| --- | --- | --- | --- | --- | --- | --- | --- | --- | --- | --- | --- |
| T3 | 5.5-10.8 | 1.95(1.63,2.32) | <0.001 | 1.43(1.25,1.64) | <0.001 | 1.21(1.05,1.40) | 0.010 | 1.27(1.09,1.48) | 0.003 | 1.60(1.48,1.74) | <0.001 |
| **TG/ HDL-C** |  |  |  |  |  |  |  |  |  |  |  |
| T1 | 0.3-2.7 | 1 | ref | 1 | ref | 1 | ref | 1 | ref | 1 | ref |
| T2 | 2.7-4.7 | 1.01(0.85,1.20) | 0.908 | 1.28(1.11,1.49) | 0.001 | 1.03(0.90,1.19) | 0.651 | 1.08(0.93,1.26) | 0.320 | 1.03(0.95,1.11) | 0.539 |
| T3 | 4.7-37 | 1.20(1.02,1.42) | 0.032 | 1.60(1.39,1.85) | <0.001 | 0.98(0.85,1.14) | 0.825 | 1.04(0.89,1.22) | 0.616 | 1.11(1.02,1.20) | 0.012 |
| **METS-IR** |  |  |  |  |  |  |  |  |  |  |  |
| T1 | 20-41.5 | 1 | ref | 1 | ref | 1 | ref | 1 | ref | 1 | ref |
| T2 | 41.5-49.1 | 0.97(0.81,1.16) | 0.771 | 1.14(0.99,1.32) | 0.065 | 1.01(0.87,1.16) | 0.920 | 1.05(0.90,1.23) | 0.504 | 0.98(0.90,1.06) | 0.612 |
| T3 | 49.1-129.1 | 1.64(1.39,1.93) | <0.001 | 1.42(1.24,1.63) | <0.001 | 1.13(0.98,1.30) | 0.096 | 1.20(1.03,1.40) | 0.021 | 1.36(1.26,1.47) | <0.001 |
| **CMI** |  |  |  |  |  |  |  |  |  |  |  |
| T1 | 0.1-0.7 | 1 | ref | 1 | ref | 1 | ref | 1 | ref | 1 | ref |
| T2 | 0.7-1.2 | 1.09(0.92,1.30) | 0.331 | 1.37(1.18,1.59) | <0.001 | 1.07(0.93,1.24) | 0.337 | 1.15(0.99,1.35) | 0.073 | 1.05(0.97,1.14) | 0.268 |
| T3 | 1.2-9.4 | 1.37(1.15,1.62) | <0.001 | 1.65(1.43,1.90) | <0.001 | 1.07(0.92,1.24) | 0.376 | 1.14(0.97,1.34) | 0.108 | 1.21(1.12,1.32) | <0.001 |
| **Model 2**  **TyG** |  |  |  |  |  |  |  |  |  |  |  |
| T1 | 6.8-8.8 | 1 | ref | 1 | ref | 1 | ref | 1 | ref | 1 | ref |
| T2 | 8.8-9.2 | 0.96(0.81,1.14) | 0.645 | 1.16(1.00,1.34) | 0.043 | 1.07(0.92,1.23) | 0.386 | 1.13(0.97,1.32) | 0.122 | 0.98(0.91,1.07) | 0.708 |
| T3 | 9.2-11.5 | 1.18(1.00,1.39) | 0.055 | 1.35(1.17,1.56) | <0.001 | 0.99(0.85,1.15) | 0.877 | 1.07(0.91,1.25) | 0.433 | 1.13(1.05,1.23) | 0.002 |
| **TyG-BMI** |  |  |  |  |  |  |  |  |  |  |  |
| T1 | 129.3-248.6 | 1 | ref | 1 | ref | 1 | ref | 1 | ref | 1 | ref |
| T2 | 248.6-287.4 | 1.07(0.90,1.28) | 0.449 | 0.99(0.86,1.13) | 0.834 | 1.03(0.89,1.18) | 0.722 | 1.02(0.87,1.19) | 0.825 | 0.99(0.91,1.08) | 0.818 |
| T3 | 287.4-715.9 | 1.56(1.32,1.85) | <0.001 | 1.25(1.09,1.43) | 0.002 | 1.02(0.89,1.19) | 0.746 | 1.05(0.90,1.23) | 0.525 | 1.27(1.17,1.37) | <0.001 |
| **TyG-WC** |  |  |  |  |  |  |  |  |  |  |  |
| T1 | 476.9-837.6 | 1 | ref | 1 | ref | 1 | ref | 1 | ref | 1 | ref |
| T2 | 837.6-946.5 | 1.23(1.02,1.48) | 0.030 | 1.20(1.04,1.39) | 0.015 | 1.00(0.86,1.16) | 0.986 | 1.01(0.86,1.19) | 0.880 | 1.11(1.02,1.21) | 0.017 |

| T3 | 946.5-1875.5 | 1.63(1.36,1.95) | <0.001 | 1.29(1.11,1.49) | 0.001 | 1.13(0.97,1.31) | 0.116 | 1.16(0.99,1.36) | 0.072 | 1.42(1.31,1.55) | <0.001 |
| --- | --- | --- | --- | --- | --- | --- | --- | --- | --- | --- | --- |
| **TyG-WHTR**  T1 | 2.8-4.9 | 1 | ref | 1 | ref | 1 | ref | 1 | ref | 1 | ref |
| T2 | 4.9-5.5 | 1.32(1.09,1.59) | 0.004 | 1.06(0.92,1.23) | 0.408 | 1.11(0.95,1.28) | 0.182 | 1.09(0.92,1.28) | 0.316 | 1.12(1.03,1.22) | 0.011 |
| T3 | 5.5-10.8 | 1.76(1.48,2.11) | <0.001 | 1.35(1.17,1.55) | <0.001 | 1.17(1.01,1.36) | 0.034 | 1.22(1.05,1.43) | 0.012 | 1.49(1.37,1.61) | <0.001 |
| **TG/ HDL-C**  T1 | 0.3-2.7 | 1 | ref | 1 | ref | 1 | ref | 1 | ref | 1 | ref |
| T2 | 2.7-4.7 | 1.01(0.85,1.20) | 0.928 | 1.28(1.11,1.49) | 0.001 | 1.04(0.90,1.20) | 0.573 | 1.09(0.93,1.27) | 0.270 | 1.03(0.95,1.12) | 0.423 |
| T3 | 4.7-37 | 1.19(1.00,1.41) | 0.048 | 1.60(1.39,1.85) | <0.001 | 0.99(0.86,1.15) | 0.933 | 1.05(0.90,1.24) | 0.520 | 1.12(1.03,1.21) | 0.009 |
| **METS-IR**  T1 | 20-41.5 | 1 | ref | 1 | ref | 1 | ref | 1 | ref | 1 | ref |
| T2 | 41.5-49.1 | 0.95(0.79,1.13) | 0.536 | 1.14(0.99,1.32) | 0.065 | 1.01(0.87,1.17) | 0.896 | 1.06(0.90,1.24) | 0.493 | 0.96(0.88,1.04) | 0.338 |
| T3 | 49.1-129.1 | 1.48(1.25,1.75) | <0.001 | 1.42(1.24,1.63) | <0.001 | 1.12(0.97,1.30) | 0.123 | 1.19(1.01,1.39) | 0.033 | 1.27(1.17,1.37) | <0.001 |
| **CMI**  T1 | 0.1-0.7 | 1 | ref | 1 | ref | 1 | ref | 1 | ref | 1 | ref |
| T2 | 0.7-1.2 | 1.08(0.91,1.29) | 0.377 | 1.37(1.18,1.59) | <0.001 | 1.08(0.94,1.25) | 0.293 | 1.16(0.99,1.36) | 0.060 | 1.05(0.97,1.14) | 0.241 |
| T3 | 1.2-9.4 | 1.32(1.11,1.56) | 0.002 | 1.65(1.43,1.90) | <0.001 | 1.07(0.92,1.24) | 0.354 | 1.15(0.97,1.35) | 0.100 | 1.20(1.10,1.30) | <0.001 |

**Note:**

Model 1: age (continuous), gender (male, female), ethnicity/race (White, Asian or Asian British, Black or Black British, Chinese, Mixed, Other ethnic group). Model 2: age (continuous), gender (male, female), ethnicity/race (White, Asian or Asian British, Black or Black British, Chinese, Mixed, Other ethnic group), educational level (less than high school, high school and above), smoking status (Yes or No), alcohol consumption (continuous), physical activity (adequate, inadequate), TC (continuous), Townsend deprivation index (three categories stratified based on tertiles), history of diabetes.

P-values less than 0.05 (p < 0.05) were considered significant.

**Abbreviations:** TyG=triglyceride-glucose, BMI=body mass index, WC= waist circumference, WHTR=waist circumference/height ratio, TG=triglyceride, HDL-C=High Density Lipoprotein-cholesterol, METSIR=Metabolic score for insulin resistance, CMI=cardiometabolic index, CVD=cardiovascular disease, HR=hazard ratio, CI=confidence interval, N=number, ref=reference.

**Table S4.** Associations of seven insulin resistance-related indices with adverse cardiovascular events calculated using Cox proportional hazards model in the patients with hyperuricemia and diabetic in UK Biobank.

**UK Biobank CVD mortality Myocardial infarction Stroke Ischemic stroke All-cause mortality**

| **TyG** | **HR (95%CI)** | **P-value** | **HR (95%CI)** | **P-value** | **HR (95%CI)** | **P-value** | **HR (95%CI)** | **P-value** | **HR (95%CI)** | **P-value** |
| --- | --- | --- | --- | --- | --- | --- | --- | --- | --- | --- |
| T1 | 1 | ref | 1 | ref | 1 | ref | 1 | ref | 1 | ref |
| T2 | 0.97(0.77,1.23) | 0.815 | 0.99(0.79,1.25) | 0.942 | 1.04(0.84,1.30) | 0.705 | 1.05(0.83,1.32) | 0.711 | 1.03(0.92,1.16) | 0.634 |
| T3 | 1.01(0.79,1.28) | 0.949 | 1.29(1.04,1.61) | 0.023 | 0.99(0.78,1.24) | 0.918 | 1.01(0.79,1.30) | 0.913 | 1.13(1.00,1.27) | 0.045 |
| **TyG-BMI**  T1 | 1 | ref | 1 | ref | 1 | ref | 1 | ref | 1 | ref |
| T2 | 1.03(0.81,1.31) | 0.814 | 1.18(0.95,1.46) | 0.146 | 0.97(0.78,1.20) | 0.753 | 0.95(0.75,1.20) | 0.674 | 1.04(0.92,1.17) | 0.539 |
| T3 | 1.34(1.06,1.71) | 0.016 | 1.26(1.01,1.58) | 0.042 | 1.01(0.80,1.26) | 0.963 | 1.02(0.80,1.31) | 0.849 | 1.34(1.19,1.50) | <0.001 |
| **TyG-WC**  T1 | 1 | ref | 1 | ref | 1 | ref | 1 | ref | 1 | ref |
| T2 | 1.25(0.97,1.61) | 0.091 | 1.23(0.97,1.55) | 0.082 | 1.03(0.82,1.30) | 0.775 | 1.03(0.80,1.31) | 0.840 | 1.14(1.01,1.29) | 0.039 |
| T3 | 1.43(1.11,1.85) | 0.006 | 1.39(1.10,1.75) | 0.006 | 1.14(0.90,1.43) | 0.283 | 1.17(0.91,1.50) | 0.227 | 1.50(1.32,1.69) | <0.001 |
| **TyG-WHTR** |  |  |  |  |  |  |  |  |  |  |

| T1 | 1 | ref | 1 | ref | 1 | ref | 1 | ref | 1 | ref |
| --- | --- | --- | --- | --- | --- | --- | --- | --- | --- | --- |
| T2 | 1.21(0.94,1.55) | 0.145 | 1.32(1.05,1.66) | 0.016 | 1.06(0.84,1.33) | 0.631 | 1.02(0.79,1.30) | 0.902 | 1.11(0.98,1.26) | 0.088 |
| T3 | 1.48(1.16,1.90) | 0.002 | 1.47(1.17,1.84) | 0.001 | 1.24(0.99,1.55) | 0.065 | 1.23(0.96,1.56) | 0.098 | 1.49(1.32,1.67) | <0.001 |
| **TG/ HDL-C**  T1 | 1 | ref | 1 | ref | 1 | ref | 1 | ref | 1 | ref |
| T2 | 1.01(0.79,1.28) | 0.957 | 1.11(0.88,1.40) | 0.364 | 0.98(0.79,1.22) | 0.851 | 0.94(0.74,1.19) | 0.608 | 1.04(0.93,1.17) | 0.485 |
| T3 | 1.02(0.80,1.30) | 0.861 | 1.27(1.01,1.59) | 0.041 | 0.95(0.75,1.19) | 0.655 | 0.93(0.73,1.18) | 0.545 | 1.04(0.92,1.17) | 0.529 |
| **METS-IR**  T1 | 1 | ref | 1 | ref | 1 | ref | 1 | ref | 1 | ref |
| T2 | 1.02(0.80,1.32) | 0.853 | 1.20(0.96,1.50) | 0.117 | 0.95(0.76,1.19) | 0.655 | 0.96(0.76,1.23) | 0.769 | 1.01(0.90,1.14) | 0.86 |
| T3 | 1.47(1.15,1.88) | 0.002 | 1.41(1.13,1.77) | 0.003 | 1.09(0.87,1.37) | 0.470 | 1.11(0.87,1.42) | 0.396 | 1.37(1.22,1.54) | <0.001 |
| **CMI**  T1 | 1 | ref | 1 | ref | 1 | ref | 1 | ref | 1 | ref |
| T2 | 0.98(0.77,1.26) | 0.899 | 1.11(0.88,1.40) | 0.387 | 0.92(0.74,1.15) | 0.455 | 0.93(0.73,1.18) | 0.548 | 1.02(0.90,1.14) | 0.781 |
| T3 | 1.22(0.96,1.55) | 0.108 | 1.41(1.12,1.76) | 0.003 | 0.97(0.78,1.22) | 0.819 | 0.96(0.75,1.23) | 0.760 | 1.18(1.05,1.33) | 0.006 |

**Note:**

Model: age (continuous), gender (male, female), ethnicity/race (White, Asian or Asian British, Black or Black British, Chinese, Mixed, Other ethnic group), educational level (less than high school, high school and above), smoking status (Yes or No), alcohol consumption (continuous), physical activity (adequate, inadequate), TC (continuous), Townsend deprivation index (three categories stratified based on tertiles), history of diabetes.

P values less than 0.05 (p < 0.05) were considered significant.

**Abbreviations:** TyG=triglyceride-glucose, BMI=body mass index, WC= waist circumference, WHTR=waist circumference/height ratio, TG=triglyceride, HDL-C=High Density Lipoprotein-cholesterol, METSIR= Metabolic score for insulin resistance, CMI=cardiometabolic index, CVD=cardiovascular disease, HR=hazard ratio, CI=confidence interval, N=number, ref=reference.

**Table S5.** Associations of seven insulin resistance-related indices with adverse cardiovascular events calculated using Cox proportional hazards model in the patients without hyperuricemia in UK Biobank.

**UK Biobank CVD mortality Myocardial infarction Stroke Ischemic stroke All-cause mortality**

| **TyG** | **HR (95%CI)** | **P-value** | **HR (95%CI)** | **P-value** | **HR (95%CI)** | **P-value** | **HR (95%CI)** | **P-value** | **HR (95%CI)** | **P-value** |
| --- | --- | --- | --- | --- | --- | --- | --- | --- | --- | --- |
| T1 | 1 | ref | 1 | ref | 1 | ref | 1 | ref | 1 | ref |
| T2 | 1.08(0.98,1.20) | 0.116 | 1.28(1.18,1.38) | <0.001 | 1.03(0.96,1.10) | 0.401 | 1.08(1.00,1.17) | 0.056 | 1.04(1.00,1.08) | 0.069 |
| T3 | 1.26(1.14,1.39) | <0.001 | 1.70(1.58,1.83) | <0.001 | 1.14(1.07,1.22) | <0.001 | 1.22(1.13,1.32) | <0.001 | 1.17(1.12,1.22) | <0.001 |
| **TyG-BMI**  T1 | 1 | ref | 1 | ref | 1 | ref | 1 | ref | 1 | ref |
| T2 | 1.15(1.04,1.27) | 0.007 | 1.28(1.18,1.38) | <0.001 | 1.07(1.00,1.15) | 0.045 | 1.11(1.02,1.20) | 0.012 | 0.95(0.92,0.99) | 0.019 |
| T3 | 1.43(1.30,1.58) | <0.001 | 1.73(1.61,1.86) | <0.001 | 1.23(1.15,1.32) | <0.001 | 1.36(1.26,1.47) | <0.001 | 1.14(1.10,1.19) | <0.001 |
| **TyG-WC**  T1 | 1 | ref | 1 | ref | 1 | ref | 1 | ref | 1 | ref |
| T2 | 1.22(1.09,1.37) | 0.001 | 1.34(1.23,1.46) | <0.001 | 1.09(1.01,1.18) | 0.020 | 1.19(1.09,1.30) | <0.001 | 1.04(1.00,1.09) | 0.061 |
| T3 | 1.64(1.46,1.84) | <0.001 | 1.79(1.64,1.94) | <0.001 | 1.26(1.16,1.36) | <0.001 | 1.41(1.29,1.54) | <0.001 | 1.26(1.21,1.32) | <0.001 |
| **TyG-WHTR** |  |  |  |  |  |  |  |  |  |  |

| T1 | 1 | ref | 1 | ref | 1 | ref | 1 | ref | 1 | ref |
| --- | --- | --- | --- | --- | --- | --- | --- | --- | --- | --- |
| T2 | 1.11(0.99,1.24) | 0.062 | 1.39(1.28,1.50) | <0.001 | 1.09(1.01,1.17) | 0.029 | 1.15(1.06,1.25) | 0.001 | 1.01(0.97,1.05) | 0.653 |
| T3 | 1.55(1.39,1.71) | <0.001 | 1.86(1.72,2.02) | <0.001 | 1.25(1.16,1.35) | <0.001 | 1.37(1.26,1.48) | <0.001 | 1.26(1.21,1.31) | <0.001 |
| **TG/ HDL-C**  T1 | 1 | ref | 1 | ref | 1 | ref | 1 | ref | 1 | ref |
| T2 | 1.08(0.98,1.20) | 0.114 | 1.37(1.27,1.49) | <0.001 | 1.03(0.96,1.11) | 0.366 | 1.08(0.99,1.16) | 0.069 | 1.04(1.00,1.09) | 0.031 |
| T3 | 1.24(1.12,1.37) | <0.001 | 1.93(1.79,2.08) | <0.001 | 1.14(1.07,1.23) | <0.001 | 1.22(1.12,1.32) | <0.001 | 1.14(1.10,1.19) | <0.001 |
| **METS-IR**  T1 | 1 | ref | 1 | ref | 1 | ref | 1 | ref | 1 | ref |
| T2 | 1.07(0.96,1.18) | 0.203 | 1.35(1.24,1.46) | <0.001 | 1.07(0.99,1.14) | 0.071 | 1.16(1.07,1.25) | <0.001 | 0.94(0.91,0.98) | 0.005 |
| T3 | 1.42(1.28,1.56) | <0.001 | 1.93(1.79,2.07) | <0.001 | 1.23(1.15,1.32) | <0.001 | 1.38(1.27,1.49) | <0.001 | 1.14(1.10,1.19) | <0.001 |
| **CMI**  T1 | 1 | ref | 1 | ref | 1 | ref | 1 | ref | 1 | ref |
| T2 | 1.11(1.00,1.23) | 0.044 | 1.47(1.35,1.59) | <0.001 | 1.04(0.97,1.12) | 0.277 | 1.09(1.01,1.19) | 0.028 | 1.05(1.01,1.10) | 0.010 |
| T3 | 1.34(1.21,1.48) | <0.001 | 2.04(1.88,2.21) | <0.001 | 1.18(1.10,1.27) | <0.001 | 1.27(1.17,1.37) | <0.001 | 1.17(1.13,1.22) | <0.001 |

**Note:**

Model: age (continuous), gender (male, female), ethnicity/race (White, Asian or Asian British, Black or Black British, Chinese, Mixed, Other ethnic group), educational level (less than high school, high school and above), smoking status (Yes or No), alcohol consumption (continuous), physical activity (adequate, inadequate), TC (continuous), Townsend deprivation index (three categories stratified based on tertiles), history of diabetes.

P-values less than 0.05 (p < 0.05) were considered significant.

**Abbreviations:** TyG=triglyceride-glucose, BMI=body mass index, WC= waist circumference, WHTR=waist circumference/height ratio, TG=triglyceride, HDL-C=High Density Lipoprotein-cholesterol, METSIR= Metabolic score for insulin resistance, CMI=cardiometabolic index, CVD=cardiovascular disease, HR=hazard ratio, CI=confidence interval, N=number, ref=reference.

**Table S6.** Associations of seven insulin resistance-related indices with adverse cardiovascular events calculated using Cox proportional hazards model based on age subgroups in UK Biobank.

| **UK Biobank** | **TyG** |  | **TyG-BMI** |  | **TyG-WC** |  | **TyG-WHTR** |  |
| --- | --- | --- | --- | --- | --- | --- | --- | --- |
| **CVD mortality**  **<65 T1** | **HR (95%CI)**  Reference | **P-value** | **HR (95%CI)**  Reference | **P-value** | **HR (95%CI)**  Reference | **P-value** | **HR (95%CI)**  Reference | **P-value** |
| **T2** | 0.86 (0.68, 1.09) |  | 0.94 (0.73, 1.21) |  | 1.04 (0.81, 1.35) |  | 1.11 (0.86, 1.43) |  |
| **T3** | 1.20 (0.96, 1.49) |  | 1.59 (1.27, 1.99) |  | 1.68 (1.33, 2.12) |  | 1.87 (1.49, 2.35) |  |
| **≥65 T1** | 2.87 (2.24, 3.66) |  | 3.11 (2.41, 4.02) |  | 2.87 (2.16, 3.81) |  | 2.69 (2.00, 3.62) |  |
| **T2** | 3.25 (2.57, 4.11) | 0.110 | 3.72 (2.90, 4.76) | 0.174 | 4.19 (3.25, 5.39) | 0.068 | 4.53 (3.54, 5.80) | 0.032 |
| **T3** | 3.32 (2.61, 4.22) | 0.844 | 4.38 (3.41, 5.63) | 0.469 | 4.62 (3.59, 5.95) | 0.823 | 4.99 (3.91, 6.38) | 0.963 |
| **Myocardial infarction**  **<65 T1** | **HR (95%CI)**  Reference | **P-value** | **HR (95%CI)**  Reference | **P-value** | **HR (95%CI)**  Reference | **P-value** | **HR (95%CI)**  Reference | **P-value** |
| **T2** | 1.19 (1.00, 1.43) |  | 1.04 (0.88, 1.23) |  | 1.20 (1.01, 1.43) |  | 1.14 (0.96, 1.36) |  |
| **T3** | 1.42 (1.20, 1.69) |  | 1.25 (1.06, 1.48) |  | 1.37 (1.15, 1.63) |  | 1.46 (1.23, 1.72) |  |
| **≥65 T1** | 1.76 (1.39, 2.22) |  | 1.82 (1.47, 2.24) |  | 1.69 (1.33, 2.15) |  | 1.83 (1.45, 2.31) |  |
| **T2** | 2.08 (1.68, 2.58) | 0.925 | 1.52 (1.21, 1.90) | 0.212 | 2.08 (1.67, 2.58) | 0.981 | 1.76 (1.41, 2.19) | 0.261 |
| **T3** | 2.08 (1.67, 2.59) | 0.237 | 2.15 (1.72, 2.67) | 0.703 | 2.03 (1.63, 2.53) | 0.397 | 2.28 (1.86, 2.81) | 0.317 |
| **All-cause mortality**  **<65 T1** | **HR (95%CI)**  Reference | **P-value** | **HR (95%CI)**  Reference | **P-value** | **HR (95%CI)**  Reference | **P-value** | **HR (95%CI)**  Reference | **P-value** |
| **T2** | 0.96 (0.86, 1.07) |  | 0.93 (0.83, 1.04) |  | 1.09 (0.97, 1.22) |  | 1.15 (1.03, 1.29) |  |
| **T3** | 1.18 (1.06, 1.31) |  | 1.25 (1.13, 1.39) |  | 1.46 (1.31, 1.62) |  | 1.63 (1.47, 1.81) |  |
| **≥65 T1** | 2.50 (2.23, 2.82) |  | 2.53 (2.25, 2.85) |  | 2.55 (2.25, 2.90) |  | 2.66 (2.34, 3.04) |  |
| **T2** | 2.74 (2.44, 3.07) | 0.150 | 2.73 (2.42, 3.07) | 0.131 | 3.02 (2.68, 3.41) | 0.416 | 3.12 (2.76, 3.52) | 0.991 |
| **T3** | 2.76 (2.46, 3.11) | 0.415 | 2.97 (2.63, 3.35) | 0.411 | 3.55 (3.15, 4.00) | 0.574 | 3.83 (3.41, 4.30) | 0.143 |
|  | **TG/ HDL-C** |  | **METSIR** |  | **CMI** |  |  |  |
| **CVD mortality** | **HR (95%CI)** | **P-value** | **HR (95%**1**C**7**I)** | **P-value** | **HR (95%CI)** | **P-value** |  |  |

| **<65 T1** | Reference |  | Reference |  | Reference |  |
| --- | --- | --- | --- | --- | --- | --- |
| **T2** | 0.92 (0.72, 1.17) |  | 0.76 (0.59, 0.98) |  | 0.94 (0.74, 1.20) |  |
| **T3** | 1.14 (0.91, 1.43) |  | 1.41 (1.13, 1.76) |  | 1.31 (1.05, 1.64) |  |
| **≥65 T1** | 2.91 (2.27, 3.73) |  | 2.86 (2.22, 3.68) |  | 2.89 (2.23, 3.73) |  |
| **T2** | 3.33 (2.63, 4.23) | 0.216 | 3.31 (2.59, 4.23) | 0.020 | 3.68 (2.90, 4.68) | 0.089 |
| **T3** | 3.25 (2.53, 4.17) | 0.892 | 3.94 (3.08, 5.05) | 0.891 | 3.56 (2.77, 4.57) | 0.718 |
| **Myocardial infarction**  **<65 T1** | **HR (95%CI)**  Reference | **P-value** | **HR (95%CI)**  Reference | **P-value** | **HR (95%CI)**  Reference | **P-value** |
| **T2** | 1.28 (1.07, 1.54) |  | 1.08 (0.91, 1.28) |  | 1.40 (1.17, 1.68) |  |
| **T3** | 1.52 (1.27, 1.81) |  | 1.37 (1.16, 1.62) |  | 1.58 (1.33, 1.89) |  |
| **≥65 T1** | 1.82 (1.44, 2.31) |  | 1.63 (1.31, 2.04) |  | 1.85 (1.45, 2.35) |  |
| **T2** | 2.11 (1.69, 2.63) | 0.607 | 1.89 (1.52, 2.34) | 0.590 | 2.24 (1.80, 2.80) | 0.351 |
| **T3** | 2.33 (1.87, 2.92) | 0.278 | 2.20 (1.76, 2.75) | 0.924 | 2.44 (1.95, 3.05) | 0.247 |
| **All-cause mortality**  **<65 T1** | **HR (95%CI)**  Reference | **P-value** | **HR (95%CI)**  Reference | **P-value** | **HR (95%CI)**  Reference | **P-value** |
| **T2** | 1.01 (0.91, 1.13) |  | 0.91 (0.82, 1.01) |  | 1.00 (0.90, 1.11) |  |
| **T3** | 1.08 (0.97, 1.20) |  | 1.22 (1.10, 1.35) |  | 1.19 (1.07, 1.32) |  |
| **≥65 T1** | 2.49 (2.22, 2.80) |  | 2.55 (2.27, 2.87) |  | 2.46 (2.18, 2.77) |  |
| **T2** | 2.76 (2.46, 3.09) | 0.404 | 2.58 (2.30, 2.91) | 0.295 | 2.88 (2.57, 3.23) | 0.102 |
| **T3** | 2.68 (2.38, 3.03) | 0.959 | 3.02 (2.68, 3.41) | 0.692 | 2.83 (2.51, 3.20) | 0.722 |

**Note:**

Model: age, gender (male, female), ethnicity/race (White, Asian or Asian British, Black or Black British, Chinese, Mixed, Other ethnic group), educational level (less than high school, high school and above), smoking status (Yes or No), alcohol consumption (continuous), physical activity (adequate, inadequate), TC (continuous), Townsend deprivation index (three categories stratified based on tertiles), history of diabetes.

P-values less than 0.05 (p < 0.05) were considered significant.

**Abbreviations:** TyG=triglyceride-glucose, BMI=body mass index, WC=waist circumference, WHTR=waist circumference/height ratio, TG=triglyceride,

HDL-C=High Density Lipoprotein-cholesterol, METSIR= Metabolic score for insulin resistance, CMI=cardiometabolic index, CVD=cardiovascular disease, HR=hazard ratio, CI=confidence interval, N=number.

**Table S7.** Associations of seven insulin resistance-related indices with adverse cardiovascular events calculated using Cox

proportional hazards model based on gender subgroups in UK Biobank.

| **UK Biobank** | **TyG** |  | **TyG- BMI** |  | **TyG-WC** |  | **TyG- WHTR** |  |
| --- | --- | --- | --- | --- | --- | --- | --- | --- |
| **CVD mortality**  **Male T1** | **HR (95%CI)**  Reference | **P-value** | **HR (95%CI)**  Reference | **P-value** | **HR (95%CI)**  Reference | **P-value** | **HR (95%CI)**  Reference | **P-value** |
| **T2** | 0.87 (0.71, 1.07) |  | 1.04 (0.85, 1.28) |  | 1.10 (0.88, 1.39) |  | 1.30 (1.04, 1.61) |  |
| **T3** | 1.15 (0.95, 1.39) |  | 1.50 (1.23, 1.82) |  | 1.47 (1.19, 1.83) |  | 1.75 (1.42, 2.16) |  |
| **Female T1** | 0.53 (0.40, 0.70) |  | 0.52 (0.38, 0.71) |  | 0.55 (0.41, 0.74) |  | 0.59 (0.43, 0.82) |  |
| **T2** | 0.62 (0.47, 0.81) | 0.114 | 0.61 (0.45, 0.83) | 0.604 | 0.74 (0.55, 1.01) | 0.209 | 0.73 (0.53, 1.00) | 0.945 |
| **T3** | 0.66 (0.50, 0.89) | 0.643 | 0.86 (0.67, 1.11) | 0.582 | 1.10 (0.81, 1.49) | 0.111 | 1.11 (0.85, 1.45) | 0.746 |
| **Myocardial infarction**  **Male T1** | **HR (95%CI)**  Reference | **P-value** | **HR (95%CI)**  Reference | **P-value** | **HR (95%CI)**  Reference | **P-value** | **HR (95%CI)**  Reference | **P-value** |
| **T2** | 1.16 (0.98, 1.37) |  | 0.99 (0.85, 1.16) |  | 1.17 (0.99, 1.40) |  | 1.07 (0.91, 1.26) |  |
| **T3** | 1.36 (1.15, 1.60) |  | 1.30 (1.11, 1.52) |  | 1.24 (1.04, 1.47) |  | 1.32 (1.13, 1.55) |  |
| **Female T1** | 0.41 (0.31, 0.52) |  | 0.42 (0.33, 0.54) |  | 0.41 (0.32, 0.52) |  | 0.39 (0.30, 0.50) |  |
| **T2** | 0.53 (0.41, 0.67) | 0.640 | 0.40 (0.31, 0.52) | 0.795 | 0.46 (0.36, 0.60) | 0.873 | 0.40 (0.31, 0.52) | 0.801 |
| **T3** | 0.54 (0.42, 0.69) | 0.885 | 0.48 (0.38, 0.60) | 0.416 | 0.62 (0.48, 0.81) | 0.220 | 0.56 (0.45, 0.70) | 0.627 |
| **All-cause mortality**  **Male T1** | **HR (95%CI)**  Reference | **P-value** | **HR (95%CI)**  Reference | **P-value** | **HR (95%CI)**  Reference | **P-value** | **HR (95%CI)**  Reference | **P-value** |
| **T2** | 0.94 (0.85, 1.04) |  | 1.00 (0.90, 1.10) |  | 1.02 (0.92, 1.14) |  | 1.12 (1.01, 1.24) |  |
| **T3** | 1.10 (1.00, 1.21) |  | 1.21 (1.09, 1.33) |  | 1.29 (1.16, 1.44) |  | 1.43 (1.29, 1.58) |  |
| **Female T1** | 0.79 (0.70, 0.90) |  | 0.80 (0.70, 0.91) |  | 0.79 (0.70, 0.90) |  | 0.81 (0.70, 0.93) |  |
| **T2** | 0.84 (0.74, 0.95) | 0.167 | 0.78 (0.68, 0.89) | 0.787 | 0.97 (0.84, 1.11) | 0.062 | 0.90 (0.78, 1.03) | 0.754 |
| **T3** | 0.98 (0.86, 1.11) | 0.176 | 1.08 (0.96, 1.21) | 0.191 | 1.30 (1.14, 1.50) | 0.005 | 1.29 (1.14, 1.45) | 0.207 |
|  | **TG/ HDL-C** |  | **METSIR** |  | **CMI** |  |  |  |

| **CVD mortality**  **Male T1** | **HR (95%CI)**  Reference | **P-value** | **HR (95%CI)**  Reference | **P-value** | **HR (95%CI)**  Reference | **P-value** |
| --- | --- | --- | --- | --- | --- | --- |
| **T2** | 0.90 (0.73, 1.11) |  | 0.87 (0.71, 1.07) |  | 1.02 (0.83, 1.26) |  |
| **T3** | 1.03 (0.85, 1.26) |  | 1.40 (1.15, 1.71) |  | 1.18 (0.96, 1.44) |  |
| **Female T1** | 0.47 (0.35, 0.61) |  | 0.50 (0.37, 0.67) |  | 0.51 (0.38, 0.68) |  |
| **T2** | 0.63 (0.47, 0.83) | 0.058 | 0.62 (0.46, 0.83) | 0.090 | 0.66 (0.50, 0.88) | 0.363 |
| **T3** | 0.75 (0.55, 1.02) | 0.022 | 0.82 (0.63, 1.07) | 0.409 | 0.85 (0.63, 1.15) | 0.066 |
| **Myocardial infarction**  **Male T1** | **HR (95%CI)**  Reference | **P-value** | **HR (95%CI)**  Reference | **P-value** | **HR (95%CI)**  Reference | **P-value** |
| **T2** | 1.29 (1.08, 1.54) |  | 1.09 (0.93, 1.29) |  | 1.40 (1.17, 1.67) |  |
| **T3** | 1.50 (1.27, 1.78) |  | 1.49 (1.27, 1.75) |  | 1.54 (1.30, 1.83) |  |
| **Female T1** | 0.46 (0.36, 0.59) |  | 0.43 (0.34, 0.55) |  | 0.45 (0.35, 0.58) |  |
| **T2** | 0.55 (0.42, 0.71) | 0.478 | 0.52 (0.40, 0.66) | 0.489 | 0.57 (0.44, 0.74) | 0.280 |
| **T3** | 0.65 (0.49, 0.85) | 0.721 | 0.51 (0.40, 0.65) | 0.161 | 0.67 (0.52, 0.88) | 0.827 |
| **All-cause mortality**  **Male T1** | **HR (95%CI)**  Reference | **P-value** | **HR (95%CI)**  Reference | **P-value** | **HR (95%CI)**  Reference | **P-value** |
| **T2** | 0.98 (0.89, 1.09) |  | 0.93 (0.84, 1.02) |  | 1.02 (0.92, 1.13) |  |
| **T3** | 1.01 (0.91, 1.11) |  | 1.19 (1.08, 1.32) |  | 1.08 (0.98, 1.20) |  |
| **Female T1** | 0.76 (0.67, 0.86) |  | 0.76 (0.67, 0.87) |  | 0.78 (0.69, 0.88) |  |
| **T2** | 0.86 (0.75, 0.98) | 0.130 | 0.81 (0.70, 0.92) | 0.200 | 0.86 (0.76, 0.99) | 0.417 |
| **T3** | 1.03 (0.89, 1.19) | 0.001 | 1.09 (0.96, 1.23) | 0.035 | 1.12 (0.98, 1.29) | 0.001 |

**Note:**

Model: age (continuous), gender (male, female), ethnicity/race (White, Asian or Asian British, Black or Black British, Chinese, Mixed, Other ethnic group), educational level (less than high school, high school and above), smoking status (Yes or No), alcohol consumption (continuous), physical activity (adequate, inadequate), TC (continuous), Townsend deprivation index (three categories stratified based on tertiles), history of diabetes.

P-values less than 0.05 (p < 0.05) were considered significant.

**Abbreviations:** TyG=triglyceride-glucose, BMI=body mass index, WC=waist circumference, WHTR=waist circumference/height ratio, TG=triglyceride, HDL-C=High Density Lipoprotein-cholesterol, METSIR= Metabolic score for insulin resistance, CMI=cardiometabolic index, CVD=cardiovascular disease, HR=hazard ratio, CI=confidence interval, N=number.

**Table S8.** Associations of seven insulin resistance-related indices with adverse cardiovascular events calculated using Cox proportional hazards models in the patients with hyperuricemia in UK Biobank.

| **UK Biobank** | **Variable** | **CVD** |  | **Myocardial** | **Stroke Ischemic All-cause** | | | | | | |
| --- | --- | --- | --- | --- | --- | --- | --- | --- | --- | --- | --- |
|  | **Range** | **mortality** |  | **infarction** |  |  |  | **stroke** |  | **mortality** |  |
| **Model 1** |  | **HR (95%CI)** | **P-value** | **HR (95%CI)** | **P-value** | **HR (95%CI)** | **P-value** | **HR (95%CI)** | **P-value** | **HR (95%CI)** | **P-value** |
| **TyG**  Q1 | 6.8-8.6 | 1 | ref | 1 | ref | 1 | ref | 1 | ref | 1 | ref |
| Q2 | 8.6-9.0 | 0.81(0.66,0.99) | 0.037 | 1.07(0.90,1.27) | 0.464 | 0.97(0.82,1.15) | 0.724 | 1.01(0.84,1.21) | 0.905 | 0.93(0.85,1.03) | 0.154 |
| Q3 | 9.0-9.4 | 1.03(0.86,1.25) | 0.726 | 1.40(1.18,1.64) | <0.001 | 1.02(0.87,1.20) | 0.808 | 1.13(0.94,1.35) | 0.183 | 1.00(0.91,1.09) | 0.958 |
| Q4 | 9.4-11.5 | 1.06(0.87,1.28) | 0.563 | 1.56(1.33,1.83) | <0.001 | 1.02(0.86,1.21) | 0.803 | 1.09(0.91,1.31) | 0.332 | 1.11(1.02,1.22) | 0.021 |
| **TyG-BMI**  Q1 | 129.3-238.8 | 1 | ref | 1 | ref | 1 | ref | 1 | ref | 1 | ref |
| Q2 | 238.8-267.1 | 0.93(0.76,1.14) | 0.511 | 1.07(0.91,1.27) | 0.387 | 0.98(0.83,1.16) | 0.847 | 0.98(0.81,1.17) | 0.787 | 0.91(0.82,1.00) | 0.040 |
| Q3 | 267.1-300.1 | 1.12(0.92,1.37) | 0.254 | 1.24(1.06,1.45) | 0.008 | 1.03(0.88,1.22) | 0.699 | 1.09(0.91,1.30) | 0.343 | 1.04(0.95,1.14) | 0.437 |
| Q4 | 300.1-715.9 | 1.67(1.38,2.01) | <0.001 | 1.44(1.23,1.69) | <0.001 | 1.07(0.91,1.27) | 0.423 | 1.10(0.92,1.32) | 0.301 | 1.39(1.27,1.52) | <0.001 |
| **TyG-WC**  Q1 | 476.9-808.1 | 1 | ref | 1 | ref | 1 | ref | 1 | ref | 1 | ref |
| Q2 | 808.1-891.1 | 1.16(0.93,1.44) | 0.201 | 1.23(1.04,1.47) | 0.017 | 1.02(0.85,1.21) | 0.858 | 1.08(0.89,1.30) | 0.424 | 1.01(0.91,1.12) | 0.854 |
| Q3 | 891.1-978.2 | 1.46(1.18,1.81) | 0.001 | 1.27(1.07,1.50) | 0.007 | 1.04(0.88,1.24) | 0.621 | 1.08(0.89,1.31) | 0.426 | 1.23(1.11,1.36) | <0.001 |
| Q4 | 978.2-1875.5 | 1.94(1.58,2.39) | <0.001 | 1.46(1.23,1.73) | <0.001 | 1.22(1.03,1.45) | 0.020 | 1.32(1.10,1.59) | 0.003 | 1.61(1.46,1.77) | <0.001 |
| **TyG-WHTR**  Q1 | 2.8-4.7 | 1 | ref | 1 | ref | 1 | ref | 1 | ref | 1 | ref |
| Q2 | 4.7-5.2 | 1.14(0.92,1.42) | 0.238 | 1.41(1.19,1.67) | <0.001 | 1.20(1.01,1.42) | 0.040 | 1.28(1.06,1.55) | 0.009 | 1.10(1.00,1.22) | 0.056 |
| Q3 | 5.2-5.7 | 1.41(1.15,1.74) | 0.001 | 1.44(1.21,1.70) | <0.001 | 1.10(0.92,1.31) | 0.289 | 1.13(0.93,1.37) | 0.203 | 1.25(1.13,1.38) | <0.001 |
| Q4 | 5.7-10.8 | 1.95(1.59,2.38) | <0.001 | 1.67(1.41,1.98) | <0.001 | 1.33(1.12,1.57) | 0.001 | 1.44(1.20,1.73) | <0.001 | 1.68(1.53,1.84) | <0.001 |

| **TG/ HDL-C** |  |  |  |  |  |  |  |  |  |  |  |
| --- | --- | --- | --- | --- | --- | --- | --- | --- | --- | --- | --- |
| Q1 | 0.3-2.3 | 1 | ref | 1 | ref | 1 | ref | 1 | ref | 1 | ref |
| Q2 | 2.3-3.6 | 0.94(0.77,1.15) | 0.543 | 1.24(1.04,1.48) | 0.018 | 0.97(0.82,1.14) | 0.717 | 1.04(0.87,1.24) | 0.67 | 0.96(0.87,1.05) | 0.364 |
| Q3 | 3.6-5.5 | 1.01(0.83,1.23) | 0.914 | 1.48(1.25,1.75) | <0.001 | 1.03(0.88,1.22) | 0.690 | 1.07(0.90,1.28) | 0.448 | 1.02(0.93,1.12) | 0.639 |
| Q4 | 5.5-37.0 | 1.16(0.96,1.41) | 0.134 | 1.72(1.46,2.03) | <0.001 | 0.96(0.81,1.14) | 0.622 | 1.04(0.87,1.25) | 0.670 | 1.08(0.99,1.19) | 0.088 |
| **METS-IR** |  |  |  |  |  |  |  |  |  |  |  |
| Q1 | 20-39.6 | 1 | ref | 1 | ref | 1 | ref | 1 | ref | 1 | ref |
| Q2 | 39.6-45.1 | 0.98(0.80,1.20) | 0.871 | 1.34(1.12,1.60) | 0.001 | 0.92(0.78,1.08) | 0.317 | 0.98(0.81,1.17) | 0.792 | 1.00(0.91,1.10) | 0.965 |
| Q3 | 45.1-51.5 | 1.02(0.84,1.25) | 0.825 | 1.49(1.25,1.77) | <0.001 | 1.00(0.85,1.18) | 0.975 | 1.10(0.92,1.32) | 0.284 | 1.06(0.96,1.16) | 0.243 |
| Q4 | 51.5-129.1 | 1.38(1.14,1.67) | 0.001 | 1.87(1.58,2.21) | <0.001 | 1.07(0.91,1.26) | 0.400 | 1.16(0.97,1.39) | 0.109 | 1.24(1.13,1.36) | <0.001 |
| **CMI** |  |  |  |  |  |  |  |  |  |  |  |
| Q1 | 0.1-0.6 | 1 | ref | 1 | ref | 1 | ref | 1 | ref | 1 | ref |
| Q2 | 0.6-0.9 | 0.99(0.81,1.22) | 0.932 | 1.10(0.93,1.30) | 0.263 | 1.02(0.87,1.21) | 0.776 | 1.14(0.95,1.36) | 0.158 | 0.94(0.85,1.03) | 0.167 |
| Q3 | 0.9-1.4 | 1.15(0.94,1.40) | 0.183 | 1.30(1.10,1.52) | 0.002 | 0.95(0.80,1.12) | 0.523 | 0.99(0.82,1.19) | 0.903 | 1.04(0.95,1.14) | 0.402 |
| Q4 | 1.4-9.4 | 1.79(1.48,2.16) | <0.001 | 1.47(1.26,1.73) | <0.001 | 1.06(0.90,1.26) | 0.474 | 1.18(0.98,1.42) | 0.074 | 1.43(1.31,1.56) | <0.001 |
| **Model 2** |  | **CVD**  **mortality** |  | **Myocardial infarction** |  | **Stroke** |  | **Ischemic stroke** |  | **All-cause mortality** |  |
|  |  | **HR (95%CI)** | **P-value** | **HR (95%CI)** | **P-value** | **HR (95%CI)** | **P-value** | **HR (95%CI)** | **P-value** | **HR (95%CI)** | **P-value** |
| **TyG** |  |  |  |  |  |  |  |  |  |  |  |
| Q1 | 6.8-8.6 | 1 | ref | 1 | ref | 1 | ref | 1 | ref | 1 | ref |
| Q2 | 8.6-9.0 | 0.82(0.67,1.01) | 0.056 | 1.02(0.86,1.21) | 0.837 | 0.97(0.82,1.15) | 0.741 | 1.02(0.85,1.22) | 0.863 | 0.95(0.87,1.04) | 0.287 |
| Q3 | 9.0-9.4 | 1.05(0.87,1.27) | 0.597 | 1.28(1.09,1.51) | 0.003 | 1.02(0.86,1.21) | 0.813 | 1.14(0.95,1.36) | 0.169 | 1.02(0.93,1.12) | 0.651 |
| Q4 | 9.4-11.5 | 1.08(0.89,1.31) | 0.457 | 1.39(1.18,1.64) | <0.001 | 1.01(0.86,1.20) | 0.871 | 1.09(0.91,1.32) | 0.341 | 1.14(1.04,1.25) | 0.007 |
| **TyG-BMI** |  |  |  |  |  |  |  |  |  |  |  |
| Q1 | 129.3-238.8 | 1 | ref | 1 | ref | 1 | ref | 1 | ref | 1 | ref |
| Q2 | 238.8-267.1 | 0.93(0.76,1.14) | 0.471 | 1.04(0.88,1.23) | 0.641 | 0.98(0.83,1.16) | 0.828 | 0.97(0.81,1.17) | 0.774 | 0.90(0.82,0.99) | 0.032 |

| Q3 | 267.1-300.1 | 1.07(0.87,1.30) | 0.527 | 1.18(1.01,1.39) | 0.042 | 1.02(0.86,1.20) | 0.851 | 1.07(0.90,1.28) | 0.452 | 1.00(0.91,1.09) | 0.963 |
| --- | --- | --- | --- | --- | --- | --- | --- | --- | --- | --- | --- |
| Q4 | 300.1-715.9 | 1.51(1.24,1.82) | <0.001 | 1.38(1.17,1.62) | <0.001 | 1.04(0.88,1.23) | 0.643 | 1.07(0.89,1.28) | 0.495 | 1.29(1.18,1.41) | <0.001 |
| **TyG-WC**  Q1 | 476.9-808.1 | 1 | ref | 1 | ref | 1 | ref | 1 | ref | 1 | ref |
| Q2 | 808.1-891.1 | 1.13(0.91,1.41) | 0.276 | 1.19(1.00,1.42) | 0.047 | 1.01(0.85,1.20) | 0.937 | 1.07(0.89,1.29) | 0.478 | 0.99(0.90,1.10) | 0.898 |
| Q3 | 891.1-978.2 | 1.39(1.12,1.72) | 0.003 | 1.20(1.01,1.43) | 0.037 | 1.03(0.86,1.22) | 0.762 | 1.06(0.88,1.29) | 0.534 | 1.18(1.07,1.30) | 0.001 |
| Q4 | 978.2-1875.5 | 1.74(1.41,2.15) | <0.001 | 1.37(1.15,1.62) | <0.001 | 1.18(0.99,1.40) | 0.063 | 1.27(1.05,1.53) | 0.012 | 1.48(1.34,1.62) | <0.001 |
| **TyG-WHTR**  Q1 | 2.8-4.7 | 1 | ref | 1 | ref | 1 | ref | 1 | ref | 1 | ref |
| Q2 | 4.7-5.2 | 1.11(0.89,1.39) | 0.332 | 1.35(1.14,1.61) | 0.001 | 1.19(1.00,1.41) | 0.052 | 1.27(1.05,1.54) | 0.012 | 1.08(0.98,1.20) | 0.124 |
| Q3 | 5.2-5.7 | 1.34(1.09,1.66) | 0.006 | 1.36(1.14,1.61) | <0.001 | 1.08(0.91,1.29) | 0.397 | 1.11(0.92,1.35) | 0.282 | 1.20(1.09,1.32) | <0.001 |
| Q4 | 5.7-10.8 | 1.73(1.41,2.12) | <0.001 | 1.56(1.31,1.84) | <0.001 | 1.28(1.08,1.52) | 0.005 | 1.38(1.15,1.67) | 0.001 | 1.54(1.40,1.69) | <0.001 |
| **TG/ HDL-C**  Q1 | 0.3-2.3 | 1 | ref | 1 | ref | 1 | ref | 1 | ref | 1 | ref |
| Q2 | 2.3-3.6 | 0.94(0.77,1.14) | 0.528 | 1.20(1.00,1.43) | 0.045 | 0.98(0.83,1.15) | 0.784 | 1.05(0.88,1.25) | 0.608 | 0.96(0.88,1.06) | 0.432 |
| Q3 | 3.6-5.5 | 1.01(0.83,1.22) | 0.945 | 1.39(1.17,1.65) | <0.001 | 1.05(0.89,1.23) | 0.590 | 1.09(0.91,1.30) | 0.371 | 1.03(0.94,1.13) | 0.482 |
| Q4 | 5.5-37.0 | 1.14(0.94,1.38) | 0.198 | 1.56(1.32,1.85) | <0.001 | 0.97(0.81,1.15) | 0.714 | 1.05(0.87,1.27) | 0.579 | 1.09(0.99,1.19) | 0.081 |
| **METS-IR**  Q1 | 20-39.6 | 1 | ref | 1 | ref | 1 | ref | 1 | ref | 1 | ref |
| Q2 | 39.6-45.1 | 0.94(0.77,1.14) | 0.528 | 1.29(1.08,1.54) | 0.005 | 0.93(0.79,1.10) | 0.382 | 0.99(0.82,1.18) | 0.884 | 1.00(0.91,1.10) | 0.945 |
| Q3 | 45.1-51.5 | 1.01(0.83,1.22) | 0.945 | 1.41(1.18,1.67) | <0.001 | 1.00(0.85,1.18) | 0.986 | 1.10(0.92,1.31) | 0.299 | 1.06(0.96,1.16) | 0.251 |
| Q4 | 51.5-129.1 | 1.14(0.94,1.38) | 0.198 | 1.70(1.43,2.01) | <0.001 | 1.06(0.90,1.26) | 0.477 | 1.14(0.95,1.37) | 0.154 | 1.22(1.11,1.34) | <0.001 |
| **CMI**  Q1 | 0.1-0.6 | 1 | ref | 1 | ref | 1 | ref | 1 | ref | 1 | ref |
| Q2 | 0.6-0.9 | 0.99(0.81,1.22) | 0.962 | 1.08(0.91,1.28) | 0.372 | 1.03(0.88,1.22) | 0.707 | 1.15(0.96,1.37) | 0.134 | 0.94(0.85,1.03) | 0.202 |
| Q3 | 0.9-1.4 | 1.09(0.89,1.33) | 0.409 | 1.27(1.08,1.49) | 0.004 | 0.95(0.81,1.13) | 0.582 | 1.00(0.83,1.20) | 0.969 | 1.00(0.91,1.10) | 0.925 |

Q4 1.4-9.4 1.61(1.32,1.95) <0.001 1.46(1.24,1.72) <0.001 1.07(0.90,1.27) 0.446 1.19(0.99,1.43) 0.067 1.33(1.21,1.45) <0.001

**Note:**

Model1: age (continuous), gender (male, female), ethnicity/race (White, Asian or Asian British, Black or Black British, Chinese, Mixed, Other ethnic group). Model2: age (continuous), gender (male, female), ethnicity/race (White, Asian or Asian British, Black or Black British, Chinese, Mixed, Other ethnic group), educational level (less than high school, high school and above), smoking status (Yes or No), alcohol consumption (continuous), physical activity (adequate, inadequate), TC (continuous), Townsend deprivation index (three categories stratified based on tertiles), history of diabetes.

P-values less than 0.05 (p < 0.05) were considered significant.

**Abbreviations:** TyG =triglyceride-glucose, BMI=body mass index, WC=waist circumference, WHTR=waist circumference/height ratio, TG=triglyceride, HDL-C=High Density Lipoprotein-cholesterol, METSIR=Metabolic score for insulin resistance, CMI=cardiometabolic index, CVD=cardiovascular disease, HR=hazard ratio, CI=confidence interval, N=number, ref=reference.

**Table S9.** Associations of seven insulin resistance-related indices with adverse cardiovascular events calculated using Cox proportional hazards model in the patients without hyperuricemia in UK Biobank.

| **UK Biobank** | **CVD** |  | **Myocardial** | **Stroke Ischemic All-cause** | | | | | | |
| --- | --- | --- | --- | --- | --- | --- | --- | --- | --- | --- |
| **TyG** | **mortality**  **HR (95%CI)** | **P-value** | **infarction**  **HR (95%CI)** | **P-value** | **HR (95%CI)** | **P-value** | **stroke**  **HR (95%CI)** | **P-value** | **mortality**  **HR (95%CI)** | **P-value** |
| Q1 | 1 | ref | 1 | ref | 1 | ref | 1 | ref | 1 | ref |
| Q2 | 1.01(0.90,1.14) | 0.810 | 1.16(1.06,1.28) | 0.002 | 0.97(0.89,1.05) | 0.490 | 1.00(0.91,1.10) | 0.951 | 1.02(0.97,1.07) | 0.480 |
| Q3 | 1.11(0.99,1.25) | 0.064 | 1.39(1.27,1.52) | <0.001 | 1.05(0.97,1.14) | 0.232 | 1.11(1.01,1.22) | 0.024 | 1.07(1.02,1.12) | 0.005 |
| Q4 | 1.26(1.13,1.41) | <0.001 | 1.81(1.66,1.97) | <0.001 | 1.15(1.06,1.25) | 0.001 | 1.24(1.13,1.35) | <0.001 | 1.19(1.14,1.25) | <0.001 |
| **TyG-BMI**  Q1 | 1 | ref | 1 | ref | 1 | ref | 1 | ref | 1 | ref |
| Q2 | 1.15(1.02,1.30) | 0.021 | 1.24(1.13,1.35) | <0.001 | 1.04(0.95,1.13) | 0.400 | 1.09(0.99,1.20) | 0.066 | 0.94(0.90,0.99) | 0.011 |
| Q3 | 1.12(0.99,1.26) | 0.064 | 1.45(1.32,1.58) | <0.001 | 1.07(0.99,1.16) | 0.091 | 1.17(1.06,1.28) | 0.001 | 0.93(0.89,0.97) | 0.002 |
| Q4 | 1.54(1.37,1.72) | <0.001 | 1.89(1.74,2.06) | <0.001 | 1.25(1.16,1.35) | <0.001 | 1.41(1.29,1.55) | <0.001 | 1.16(1.11,1.21) | <0.001 |
| **TyG-WC**  Q1 | 1 | ref | 1 | ref | 1 | ref | 1 | ref | 1 | ref |
| Q2 | 1.19(1.04,1.37) | 0.013 | 1.34(1.20,1.48) | <0.001 | 1.16(1.06,1.26) | 0.001 | 1.25(1.13,1.39) | <0.001 | 1.03(0.98,1.08) | 0.245 |
| Q3 | 1.29(1.12,1.48) | <0.001 | 1.65(1.49,1.83) | <0.001 | 1.15(1.05,1.26) | 0.002 | 1.30(1.17,1.45) | <0.001 | 1.07(1.01,1.12) | 0.015 |
| Q4 | 1.77(1.54,2.02) | <0.001 | 2.07(1.87,2.30) | <0.001 | 1.34(1.22,1.46) | <0.001 | 1.52(1.37,1.69) | <0.001 | 1.31(1.25,1.38) | <0.001 |
| **TyG-WHTR**  Q1 | 1 | ref | 1 | ref | 1 | ref | 1 | ref | 1 | ref |
| Q2 | 1.22(1.07,1.39) | 0.004 | 1.37(1.24,1.52) | <0.001 | 1.14(1.04,1.24) | 0.004 | 1.22(1.11,1.36) | <0.001 | 1.03(0.98,1.08) | 0.294 |
| Q3 | 1.27(1.11,1.45) | <0.001 | 1.70(1.54,1.88) | <0.001 | 1.12(1.02,1.22) | 0.015 | 1.25(1.13,1.39) | <0.001 | 1.04(0.99,1.10) | 0.093 |
| Q4 | 1.77(1.56,2.01) | <0.001 | 2.14(1.95,2.36) | <0.001 | 1.36(1.25,1.48) | <0.001 | 1.56(1.41,1.72) | <0.001 | 1.33(1.26,1.39) | <0.001 |

| **TG/ HDL-C**  Q1 | 1 | ref | 1 | ref | 1 | ref | 1 | ref | 1 | ref |
| --- | --- | --- | --- | --- | --- | --- | --- | --- | --- | --- |
| Q2 | 1.01(0.90,1.14) | 0.838 | 1.38(1.25,1.52) | <0.001 | 1.07(0.99,1.17) | 0.084 | 1.12(1.02,1.23) | 0.015 | 1.02(0.98,1.07) | 0.328 |
| Q3 | 1.15(1.03,1.29) | 0.015 | 1.66(1.51,1.82) | <0.001 | 1.04(0.96,1.13) | 0.313 | 1.12(1.02,1.23) | 0.019 | 1.06(1.01,1.11) | 0.016 |
| Q4 | 1.26(1.13,1.41) | <0.001 | 2.20(2.01,2.41) | <0.001 | 1.21(1.12,1.31) | <0.001 | 1.29(1.18,1.42) | <0.001 | 1.17(1.11,1.22) | <0.001 |
| **METS-IR**  Q1 | 1 | ref | 1 | ref | 1 | ref | 1 | ref | 1 | ref |
| Q2 | 1.05(0.93,1.19) | 0.405 | 1.28(1.16,1.40) | <0.001 | 1.05(0.96,1.14) | 0.275 | 1.12(1.02,1.23) | 0.021 | 0.92(0.87,0.96) | <0.001 |
| Q3 | 1.13(1.00,1.27) | 0.050 | 1.60(1.46,1.76) | <0.001 | 1.10(1.02,1.20) | 0.018 | 1.22(1.11,1.34) | <0.001 | 0.93(0.89,0.97) | 0.002 |
| Q4 | 1.52(1.36,1.71) | <0.001 | 2.13(1.95,2.33) | <0.001 | 1.26(1.16,1.36) | <0.001 | 1.42(1.29,1.56) | <0.001 | 1.16(1.11,1.21) | <0.001 |
| **CMI**  Q1 | 1 | ref | 1 | ref | 1 | ref | 1 | ref | 1 | ref |
| Q2 | 1.06(0.93,1.20) | 0.378 | 1.46(1.32,1.62) | <0.001 | 1.09(1.00,1.19) | 0.039 | 1.16(1.06,1.28) | 0.002 | 1.04(0.99,1.09) | 0.120 |
| Q3 | 1.19(1.06,1.34) | 0.004 | 1.78(1.61,1.96) | <0.001 | 1.10(1.01,1.20) | 0.021 | 1.20(1.09,1.32) | <0.001 | 1.11(1.06,1.16) | <0.001 |
| Q4 | 1.41(1.26,1.59) | <0.001 | 2.40(2.18,2.64) | <0.001 | 1.24(1.15,1.35) | <0.001 | 1.36(1.24,1.49) | <0.001 | 1.22(1.16,1.27) | <0.001 |

**Note:**

Model: age (continuous), gender (male, female), ethnicity/race (White, Asian or Asian British, Black or Black British, Chinese, Mixed, Other ethnic group), educational level (less than high school, high school and above), smoking status (Yes or No), alcohol consumption (continuous), physical activity (adequate, inadequate), TC (continuous), Townsend deprivation index (three categories stratified based on tertiles), history of diabetes.

P-values less than 0.05 (p < 0.05) were considered significant.

**Abbreviations:** TyG=triglyceride-glucose, BMI=body mass index, WC=waist circumference, WHTR=waist circumference/height ratio, TG=triglyceride, HDL-C=High Density Lipoprotein-cholesterol, METSIR=Metabolic score for insulin resistance, CMI=cardiometabolic index, CVD=cardiovascular disease, HR=hazard ratio, CI=confidence interval, N=number, ref=reference.

**Table S10.** Associations of seven insulin resistance-related indices with adverse cardiovascular events calculated using Cox proportional hazards model in the patients with hyperuricemia and diabetes in UK Biobank.

| **UK Biobank** | **CVD** |  | **Myocardial** | **Stroke Ischemic All-cause** | | | | | | |
| --- | --- | --- | --- | --- | --- | --- | --- | --- | --- | --- |
| **TyG** | **mortality**  **HR (95%CI)** | **P-value** | **infarction**  **HR (95%CI)** | **P-value** | **HR (95%CI)** | **P-value** | **stroke**  **HR (95%CI)** | **P-value** | **mortality**  **HR (95%CI)** | **P-value** |
| Q1 | 1 | ref | 1 | ref | 1 | ref | 1 | ref | 1 | ref |
| Q2 | 1.24(0.81,1.90) | 0.312 | 1.20(0.76,1.88) | 0.439 | 1.29(0.83,2.00) | 0.258 | 1.37(0.86,2.18) | 0.184 | 0.97(0.78,1.22) | 0.826 |
| Q3 | 1.16(0.74,1.80) | 0.522 | 1.34(0.86,2.11) | 0.196 | 1.05(0.65,1.68) | 0.852 | 1.08(0.65,1.78) | 0.775 | 1.14(0.91,1.43) | 0.262 |
| Q4 | 0.90(0.55,1.47) | 0.681 | 1.37(0.87,2.18) | 0.178 | 1.27(0.79,2.03) | 0.330 | 1.27(0.77,2.11) | 0.352 | 0.97(0.76,1.24) | 0.811 |
| **TyG-BMI**  Q1 | 1 | ref | 1 | ref | 1 | ref | 1 | ref | 1 | ref |
| Q2 | 1.14(0.73,1.79) | 0.568 | 1.39(0.91,2.13) | 0.131 | 1.17(0.75,1.83) | 0.490 | 1.08(0.68,1.73) | 0.736 | 1.05(0.83,1.32) | 0.677 |
| Q3 | 1.18(0.75,1.87) | 0.474 | 1.28(0.82,1.99) | 0.275 | 0.92(0.57,1.48) | 0.724 | 0.87(0.53,1.44) | 0.589 | 1.13(0.90,1.43) | 0.305 |
| Q4 | 1.65(1.04,2.61) | 0.033 | 1.06(0.65,1.73) | 0.808 | 1.55(0.98,2.44) | 0.061 | 1.47(0.92,2.37) | 0.109 | 1.42(1.12,1.80) | 0.004 |
| **TyG-WC**  Q1 | 1 | ref | 1 | ref | 1 | ref | 1 | ref | 1 | ref |
| Q2 | 1.20(0.74,1.97) | 0.458 | 1.15(0.74,1.81) | 0.531 | 1.28(0.79,2.07) | 0.320 | 1.29(0.77,2.13) | 0.331 | 1.00(0.78,1.27) | 0.978 |
| Q3 | 1.35(0.84,2.20) | 0.218 | 1.15(0.73,1.80) | 0.557 | 1.30(0.79,2.13) | 0.298 | 1.25(0.74,2.10) | 0.399 | 1.13(0.89,1.44) | 0.315 |
| Q4 | 1.69(1.04,2.75) | 0.034 | 0.98(0.61,1.58) | 0.939 | 2.05(1.27,3.28) | 0.003 | 1.98(1.20,3.26) | 0.007 | 1.55(1.22,1.97) | <0.001 |
| **TyG-WHTR**  Q1 | 1 | ref | 1 | ref | 1 | ref | 1 | ref | 1 | ref |
| Q2 | 1.99(1.20,3.29) | 0.007 | 1.51(0.98,2.33) | 0.061 | 1.57(0.96,2.57) | 0.072 | 1.42(0.85,2.37) | 0.177 | 1.23(0.97,1.57) | 0.094 |
| Q3 | 1.92(1.15,3.20) | 0.012 | 1.41(0.91,2.21) | 0.128 | 1.40(0.84,2.33) | 0.199 | 1.35(0.80,2.29) | 0.257 | 1.37(1.08,1.74) | 0.009 |
| Q4 | 2.59(1.56,4.31) | <0.001 | 1.04(0.64,1.70) | 0.865 | 2.45(1.52,3.96) | <0.001 | 2.21(1.34,3.63) | 0.002 | 1.60(1.26,2.03) | <0.001 |

| **TG/ HDL-C**  Q1 | 1 | ref | 1 | ref | 1 | ref | 1 | ref | 1 | ref |
| --- | --- | --- | --- | --- | --- | --- | --- | --- | --- | --- |
| Q2 | 1.12(0.72,1.75) | 0.620 | 1.05(0.67,1.67) | 0.826 | 1.23(0.79,1.90) | 0.364 | 1.19(0.76,1.87) | 0.453 | 1.02(0.81,1.28) | 0.858 |
| Q3 | 1.17(0.75,1.83) | 0.489 | 1.40(0.90,2.16) | 0.131 | 1.17(0.75,1.84) | 0.489 | 1.05(0.66,1.69) | 0.825 | 1.04(0.83,1.31) | 0.734 |
| Q4 | 1.08(0.67,1.72) | 0.759 | 1.05(0.66,1.67) | 0.837 | 1.07(0.66,1.72) | 0.779 | 0.93(0.56,1.53) | 0.768 | 1.04(0.82,1.31) | 0.771 |
| **METS-IR**  Q1 | 1 | ref | 1 | ref | 1 | ref | 1 | ref | 1 | ref |
| Q2 | 0.93(0.57,1.49) | 0.750 | 1.28(0.83,1.98) | 0.260 | 1.23(0.78,1.94) | 0.365 | 1.22(0.76,1.96) | 0.414 | 0.96(0.76,1.22) | 0.736 |
| Q3 | 1.20(0.76,1.89) | 0.428 | 1.15(0.73,1.79) | 0.549 | 1.08(0.67,1.73) | 0.756 | 1.12(0.68,1.82) | 0.657 | 1.15(0.92,1.45) | 0.227 |
| Q4 | 1.68(1.06,2.65) | 0.027 | 1.09(0.68,1.76) | 0.721 | 1.48(0.93,2.36) | 0.101 | 1.42(0.86,2.32) | 0.169 | 1.41(1.11,1.78) | 0.005 |
| **CMI**  Q1 | 1 | ref | 1 | ref | 1 | ref | 1 | ref | 1 | ref |
| Q2 | 1.28(0.82,1.98) | 0.275 | 1.12(0.71,1.75) | 0.630 | 1.06(0.67,1.66) | 0.807 | 1.02(0.64,1.63) | 0.926 | 0.99(0.78,1.25) | 0.919 |
| Q3 | 1.09(0.69,1.74) | 0.702 | 1.30(0.84,2.01) | 0.236 | 1.28(0.82,1.99) | 0.269 | 1.18(0.74,1.87) | 0.484 | 1.12(0.89,1.41) | 0.320 |
| Q4 | 1.21(0.76,1.92) | 0.424 | 1.02(0.64,1.62) | 0.938 | 1.12(0.70,1.79) | 0.643 | 0.98(0.60,1.61) | 0.943 | 1.11(0.87,1.40) | 0.403 |

**Note:**

Model: age (continuous), gender (male, female), ethnicity/race (White, Asian or Asian British, Black or Black British, Chinese, Mixed, Other ethnic group), educational level (less than high school, high school and above), smoking status (Yes or No), alcohol consumption (continuous), physical activity (adequate, inadequate), TC (continuous), Townsend deprivation index (three categories stratified based on tertiles), history of diabetes.

P-values less than 0.05 (p < 0.05) were considered significant.

**Abbreviations:** TyG =triglyceride-glucose, BMI=body mass index, WC=waist circumference, WHTR=waist circumference/height ratio, TG=triglyceride, HDL-C=High Density Lipoprotein-cholesterol, METSIR=Metabolic score for insulin resistance, CMI= cardiometabolic index, CVD=cardiovascular disease, HR=hazard ratio, CI=confidence interval, N=number, ref=reference.

**Table S11.** Associations of seven insulin resistance-related indices with adverse cardiovascular events calculated using Cox proportional hazards model in the patients with hyperuricemia in UK Biobank.

|  | **CVD Mortality** | | **Myocardial** infarction | |
| --- | --- | --- | --- | --- |
|  | **HR** (95%CI) | **P-value** | **HR** (95%CI) | **P-value** |
| **TyG** |  |  |  |  |
| T1 | 1 | ref | 1 | ref |
| T2 | 0.96 (0.81,1.14) | 0.645 | 1.16 (1.00,1.34) | 0.043 |
| T3 | 1.18 (1.00,1.39) | 0.055 | 1.35 (1.17,1.56) | <0.001 |
| **TyG-BMI** |  |  |  |  |
| T1 | 1 | ref | 1 | ref |
| T2 | 1.07 (0.90,1.28) | 0.449 | 0.99 (0.86,1.13) | 0.834 |
| T3 | 1.56 (1.32,1.85) | <0.001 | 1.25 (1.09,1.43) | 0.002 |
| **TyG-WC** |  |  |  |  |
| T1 | 1 | ref | 1 | ref |
| T2 | 1.23 (1.02,1.48) | 0.03 | 1.20 (1.04,1.39) | 0.015 |
| T3 | 1.63 (1.36,1.95) | <0.001 | 1.29 (1.11,1.49) | 0.001 |
| **TyG-**WHTR |  |  |  |  |
| T1 | 1 | ref | 1 | ref |
| T2 | 1.32 (1.09,1.59) | 0.004 | 1.06 (0.92,1.23) | 0.408 |
| T3 | 1.76 (1.48,2.11) | <0.001 | 1.35 (1.17,1.55) | <0.001 |
| **TG/** HDL-C |  |  |  |  |
| T1 | 1 | ref | 1 | ref |
| T2 | 1.01 (0.85,1.20) | 0.928 | 1.23 (1.06,1.43) | 0.006 |
| T3 | 1.19 (1.00,1.41) | 0.048 | 1.47 (1.27,1.70) | <0.001 |
| **METS-**IR |  |  |  |  |
| T1 | 1 | ref | 1 | ref |
| T2 | 0.95 (0.79,1.13) | 0.536 | 1.12 (0.97,1.29) | 0.109 |
| T3 | 1.48 (1.25,1.75) | <0.001 | 1.41 (1.23,1.62) | <0.001 |
| **CMI** |  |  |  |  |
| T1 | 1 | ref | 1 | ref |
| T2 | 1.08 (0.91,1.29) | 0.377 | 1.31 (1.13,1.52) | <0.001 |
| T3 | 1.32 (1.11,1.56) | 0.002 | 1.52 (1.31,1.76) | <0.001 |

**Note:** Model: age (continuous), gender (male, female), ethnicity/race (White, Asian or Asian British, Black or Black British, Chinese, Mixed, Other ethnic group), educational level (less than high school, high school and above), smoking status (Yes or No), alcohol consumption (continuous), physical activity (adequate, inadequate), TC (continuous), Townsend deprivation index (three categories stratified based on tertiles), history of diabetes, history of hypertension, LDL-C, Antihypertensive drugs, Antidiabetic drugs and Antilipemic drugs.

P values less than 0.05 (p < 0.05) were considered significant.

**Abbreviations:** TyG=triglyceride-glucose, BMI=body mass index, WC=waist circumference, WHTR=waist circumference/height ratio, TG=triglyceride, HDL-C=High Density Lipoprotein-cholesterol, METSIR= Metabolic score for insulin resistance, CMI= cardiometabolic index, CVD=cardiovascular disease, HR=hazard ratio, CI=confidence interval, N=number, ref=reference.

**Table S12.** Subdistribution hazard ratios (sHR) and 95% confidence intervals (95%CI) estimated by the Fine-Gray competing risks model.

| **Variable** | **sHR (95%CI)** | **P-value** |
| --- | --- | --- |
| **TyG** | 1.133 (0.987, 1.300) | 0.077 |
| **TyG-BMI** | 1.004 (1.003, 1.006) | <0.001 |
| **TyG-WC** | 1.002 (1.001, 1.002) | <0.001 |
| **TyG-WHTR** | 1.365 (1.245, 1.497) | <0.001 |
| **TG/HDL** | 1.023 (1.001, 1.045) | 0.039 |
| **CMI** | 1.120 (1.041, 1.205) | 0.002 |
| **METS-IR** | 1.024 (1.016, 1.032) | <0.001 |

**Note:** Model: age (continuous), gender (male, female), ethnicity/race (White, Asian or Asian British, Black or Black British, Chinese, Mixed, Other ethnic group), educational level (less than high school, high school and above), smoking status (Yes or No), alcohol consumption (continuous), physical activity (adequate, inadequate), TC (continuous), Townsend deprivation index (three categories stratified based on tertiles), history of diabetes.

P values less than 0.05 (p < 0.05) were considered significant.

**Abbreviations:** TyG=triglyceride-glucose, BMI=body mass index, WC=waist circumference, WHTR=waist circumference/height ratio, TG=triglyceride, HDL-C=High Density Lipoprotein- cholesterol, METSIR= Metabolic score for insulin resistance, CMI=cardiometabolic index, CVD=cardiovascular disease, HR=hazard ratio, CI=confidence interval, N=number, ref=reference.

**Table S13.** MR-PRESSO Global Pleiotropy Test and Outlier Count.

| Exposure | Outcome | Global P-value | Has Outlier SNPs | Has Outlier SNPs |
| --- | --- | --- | --- | --- |
| European | | | | |
| Fasting glucose | Angina pectoris | 6.00E-04 | FALSE | 0 |
| Fasting glucose | Heart failure and coronary heart disease | 0.3332 | FALSE | 0 |
| Fasting glucose | Myocardial infarction | 0.0084 | FALSE | 0 |
| Fasting glucose | Embolic stroke | 0.4911 | FALSE | 0 |
| HDL cholesterol levels | Angina pectoris | <1e-04 | FALSE | 0 |
| HDL cholesterol levels | Heart failure and coronary heart disease | <1e-04 | FALSE | 0 |
| HDL cholesterol levels | Myocardial infarction | <1e-04 | FALSE | 0 |
| HDL cholesterol levels | Embolic stroke | 0.3441 | FALSE | 0 |
| LDL cholesterol levels | Angina pectoris | <1e-04 | FALSE | 0 |
| LDL cholesterol levels | Heart failure and coronary heart disease | <1e-04 | FALSE | 0 |
| LDL cholesterol levels | Myocardial infarction | <1e-04 | FALSE | 0 |
| LDL cholesterol levels | Embolic stroke | 0.5845 | FALSE | 0 |
| Total cholesterol levels | Angina pectoris | <1e-04 | FALSE | 0 |
| Total cholesterol levels | Heart failure and coronary heart disease | <1e-04 | FALSE | 0 |
| Total cholesterol levels | Myocardial infarction | <1e-04 | FALSE | 0 |
| Total cholesterol levels | Embolic stroke | 0.1518 | FALSE | 0 |
| Total triglycerides levels | Angina pectoris | <1e-04 | FALSE | 0 |
| Total triglycerides levels | Heart failure and coronary heart disease | <1e-04 | FALSE | 0 |
| Total triglycerides levels | Myocardial infarction | <1e-04 | FALSE | 0 |
| Total triglycerides levels | Embolic stroke | 0.117 | FALSE | 0 |
| East Asian | | | | |
| HDL cholesterol | Angina pectoris | <0.001 | FALSE | 0 |
| HDL cholesterol | Myocardial infarction | <0.001 | FALSE | 0 |
| HDL cholesterol | Ischemic stroke | <0.001 | FALSE | 0 |
| HDL cholesterol | Chronic heart failure | 0.159 | FALSE | 0 |
| LDL cholesterol | Angina pectoris | <0.001 | FALSE | 0 |
| LDL cholesterol | Myocardial infarction | <0.001 | FALSE | 0 |
| LDL cholesterol | Ischemic stroke | <0.001 | FALSE | 0 |
| LDL cholesterol | Chronic heart failure | <0.001 | FALSE | 0 |
| Serum uric acid levels | Angina pectoris | <0.001 | FALSE | 0 |
| Serum uric acid levels | Myocardial infarction | <0.001 | FALSE | 0 |
| Serum uric acid levels | Ischemic stroke | <0.001 | FALSE | 0 |
| Serum uric acid levels | Chronic heart failure | <0.001 | FALSE | 0 |
| Total cholesterol levels | Angina pectoris | <0.001 | FALSE | 0 |
| Total cholesterol levels | Myocardial infarction | <0.001 | FALSE | 0 |
| Total cholesterol levels | Ischemic stroke | 0.01 | FALSE | 0 |
| Total cholesterol levels | Chronic heart failure | 0.005 | FALSE | 0 |
| Triglycerides | Angina pectoris | <0.001 | FALSE | 0 |
| Triglycerides | Myocardial infarction | <0.001 | FALSE | 0 |
| Triglycerides | Ischemic stroke | 0.035 | FALSE | 0 |
| Triglycerides | Chronic heart failure | 0.362 | FALSE | 0 |

**Table S14.** Steiger Causal Directionality Test and Instrumental Variable Filtering Results.

| Exposure | Outcome | SNP R² (Exposure) | SNP R² (Outcome) | Correct Causal Direction | Steiger P-value |
| --- | --- | --- | --- | --- | --- |
| European | | | | | |
| Fasting glucose | Angina pectoris | 0.035721 | 0.000139 | TRUE | 2.86E-298 |
| Fasting glucose | Heart failure and coronary heart disease | 0.035721 | 7.00E-05 | TRUE | 0 |
| Fasting glucose | Myocardial infarction | 0.035721 | 0.000114 | TRUE | 5.81E-302 |
| Fasting glucose | Embolic stroke | 0.035721 | 6.23E-05 | TRUE | 0 |
| HDL cholesterol levels | Angina pectoris | 0.168572 | 0.001756 | TRUE | 0 |
| HDL cholesterol levels | Heart failure and coronary heart disease | 0.168572 | 0.001004 | TRUE | 0 |
| HDL cholesterol levels | Myocardial infarction | 0.168572 | 0.001558 | TRUE | 0 |
| HDL cholesterol levels | Embolic stroke | 0.168572 | 0.000515 | TRUE | 0 |
| LDL cholesterol levels | Angina pectoris | 0.146736 | 0.002751 | TRUE | 0 |
| LDL cholesterol levels | Heart failure and coronary heart disease | 0.146736 | 0.001143 | TRUE | 0 |
| LDL cholesterol levels | Myocardial infarction | 0.146736 | 0.002159 | TRUE | 0 |
| LDL cholesterol levels | Embolic stroke | 0.146736 | 0.000351 | TRUE | 0 |
| Total cholesterol levels | Angina pectoris | 0.16511 | 0.002626 | TRUE | 0 |
| Total cholesterol levels | Heart failure and coronary heart disease | 0.16511 | 0.001169 | TRUE | 0 |
| Total cholesterol levels | Myocardial infarction | 0.16511 | 0.002225 | TRUE | 0 |
| Total cholesterol levels | Embolic stroke | 0.16511 | 0.000436 | TRUE | 0 |
| Total triglycerides levels | Angina pectoris | 0.147618 | 0.002814 | TRUE | 0 |
| Total triglycerides levels | Heart failure and coronary heart disease | 0.147618 | 0.001143 | TRUE | 0 |
| Total triglycerides levels | Myocardial infarction | 0.147618 | 0.002239 | TRUE | 0 |
| Total triglycerides levels | Embolic stroke | 0.147618 | 0.000445 | TRUE | 0 |
| East Asian | | | | | |
| HDL cholesterol | Angina pectoris | 0.190587 | 0.003332 | TRUE | 0 |
| HDL cholesterol | Myocardial infarction | 0.190587 | 0.007047 | TRUE | 0 |
| HDL cholesterol | Ischemic stroke | 0.189624 | 0.001949 | TRUE | 0 |
| HDL cholesterol | Chronic heart failure | 0.190587 | 0.001376 | TRUE | 0 |
| LDL cholesterol | Angina pectoris | 0.098864 | 0.003835 | TRUE | 0 |
| LDL cholesterol | Myocardial infarction | 0.098864 | 0.009401 | TRUE | 0 |
| LDL cholesterol | Ischemic stroke | 0.098864 | 0.001236 | TRUE | 0 |
| LDL cholesterol | Chronic heart failure | 0.098864 | 0.001074 | TRUE | 0 |
| Serum uric acid levels | Angina pectoris | 0.16094 | 0.003866 | TRUE | 0 |
| Serum uric acid levels | Myocardial infarction | 0.16094 | 0.008194 | TRUE | 0 |
| Serum uric acid levels | Ischemic stroke | 0.160768 | 0.002503 | TRUE | 0 |
| Serum uric acid levels | Chronic heart failure | 0.16094 | 0.002241 | TRUE | 0 |
| Total cholesterol levels | Angina pectoris | 0.070549 | 0.004311 | TRUE | 0 |
| Total cholesterol levels | Myocardial infarction | 0.070549 | 0.007844 | TRUE | 0 |
| Total cholesterol levels | Ischemic stroke | 0.070549 | 0.001188 | TRUE | 0 |
| Total cholesterol levels | Chronic heart failure | 0.070549 | 0.0012 | TRUE | 0 |
| Triglycerides | Angina pectoris | 0.104487 | 0.003224 | TRUE | 0 |
| Triglycerides | Myocardial infarction | 0.104487 | 0.005502 | TRUE | 0 |
| Triglycerides | Ischemic stroke | 0.103978 | 0.001226 | TRUE | 0 |
| Triglycerides | Chronic heart failure | 0.104487 | 0.001076 | TRUE | 0 |


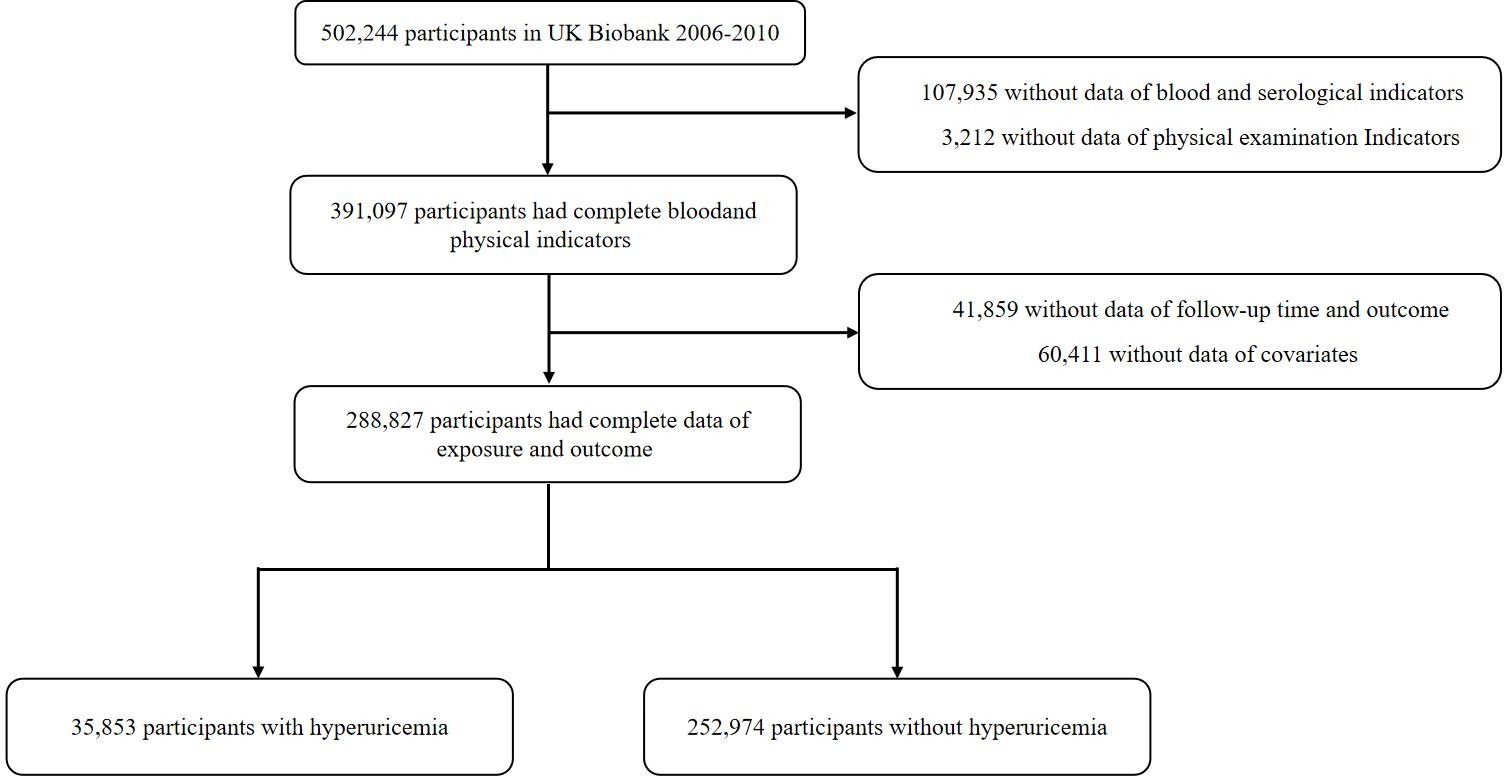


**Figure S1.** The flow chart of participants in the UK Biobank (UKBB) cohort.


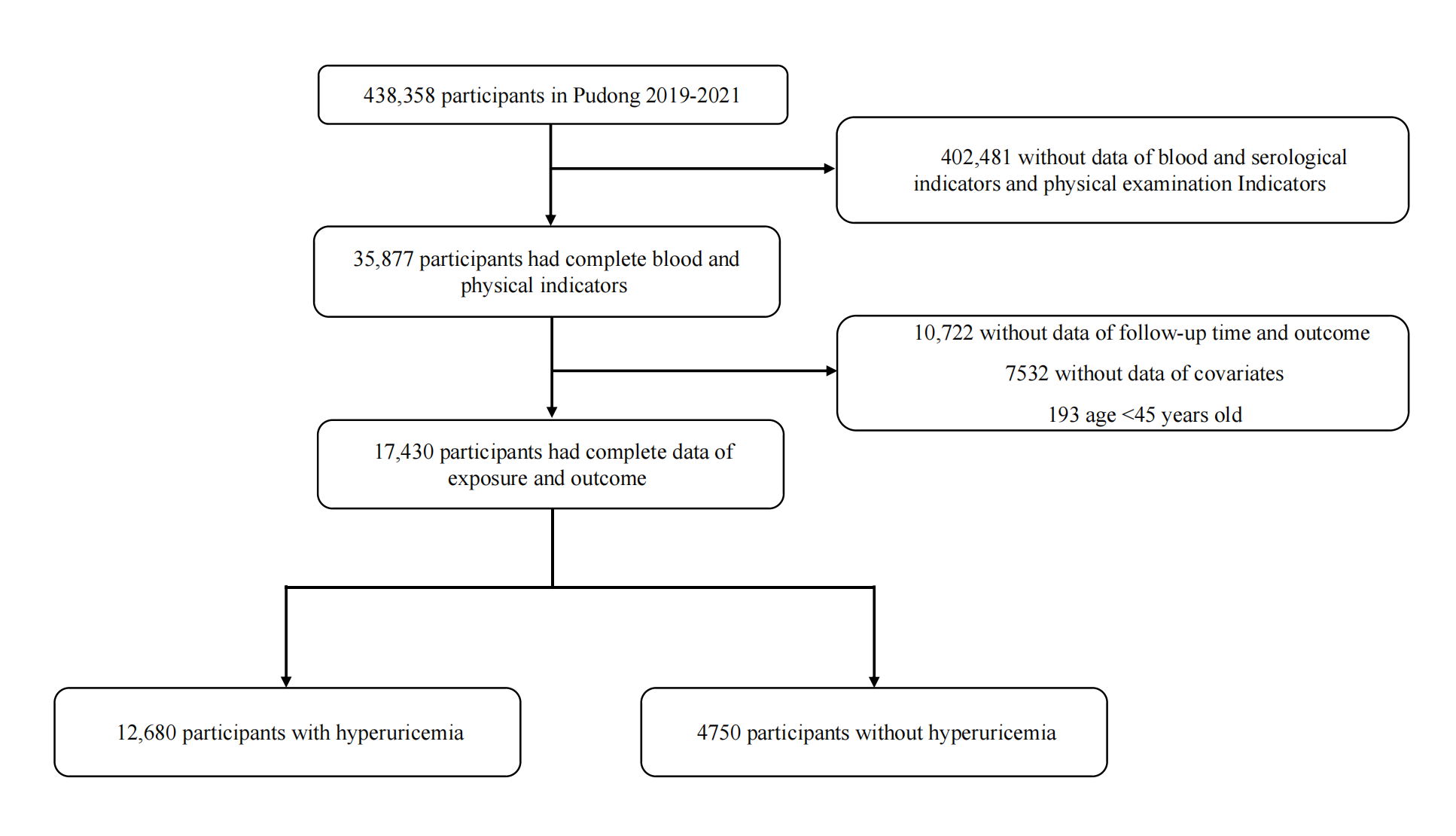


**Figure S2.** The flow chart of participants in the Shanghai Pudong cohort.


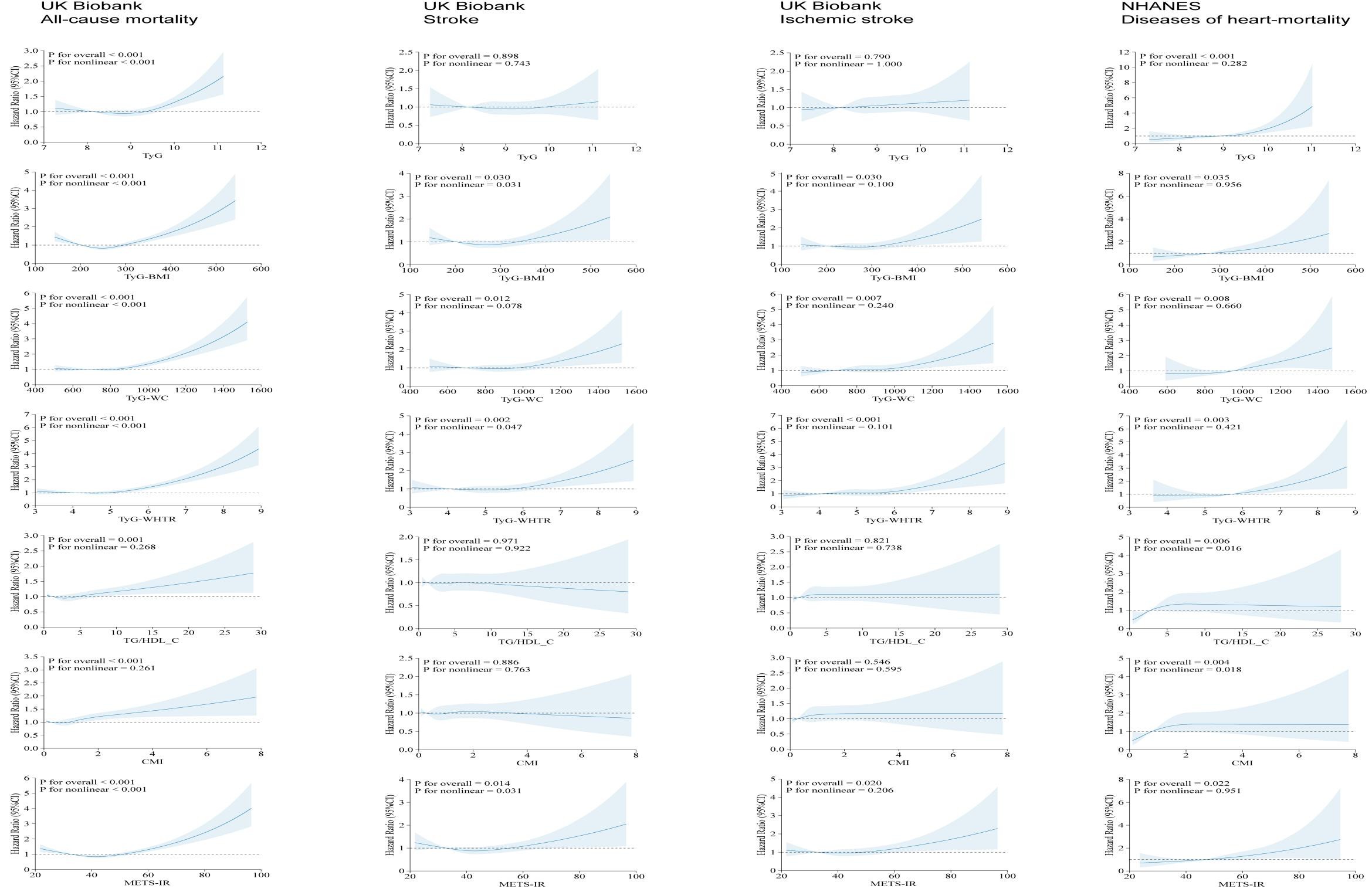


**Figure S3.** Restricted cubic spline (RCS) curve illustrating the associations of insulin resistance (IR)-related indices with adverse cardiovascular events.

**Note:** UKBB: Model was adjusted by age (continuous), sex (male, female), ethnicity/race (White, Asian or Asian British, Black or Black British,

Chinese, Mixed, Other ethnic group), educational level (less than high school, high school and above), smoking status (Yes or No), alcohol consumption (continuous), physical activity (adequate, inadequate), TC (continuous), Townsend deprivation index (three categories stratified based on tertiles), history of diabetes.

Abbreviations: TyG=triglyceride-glucose, BMI=body mass index, WC=waist circumference, WHTR=waist circumference/height ratio, TG=triglyceride, HDL-C=High Density Lipoprotein-cholesterol, METSIR= Metabolic score for insulin resistance, CMI=cardiometabolic index, CVD=cardiovascular disease.


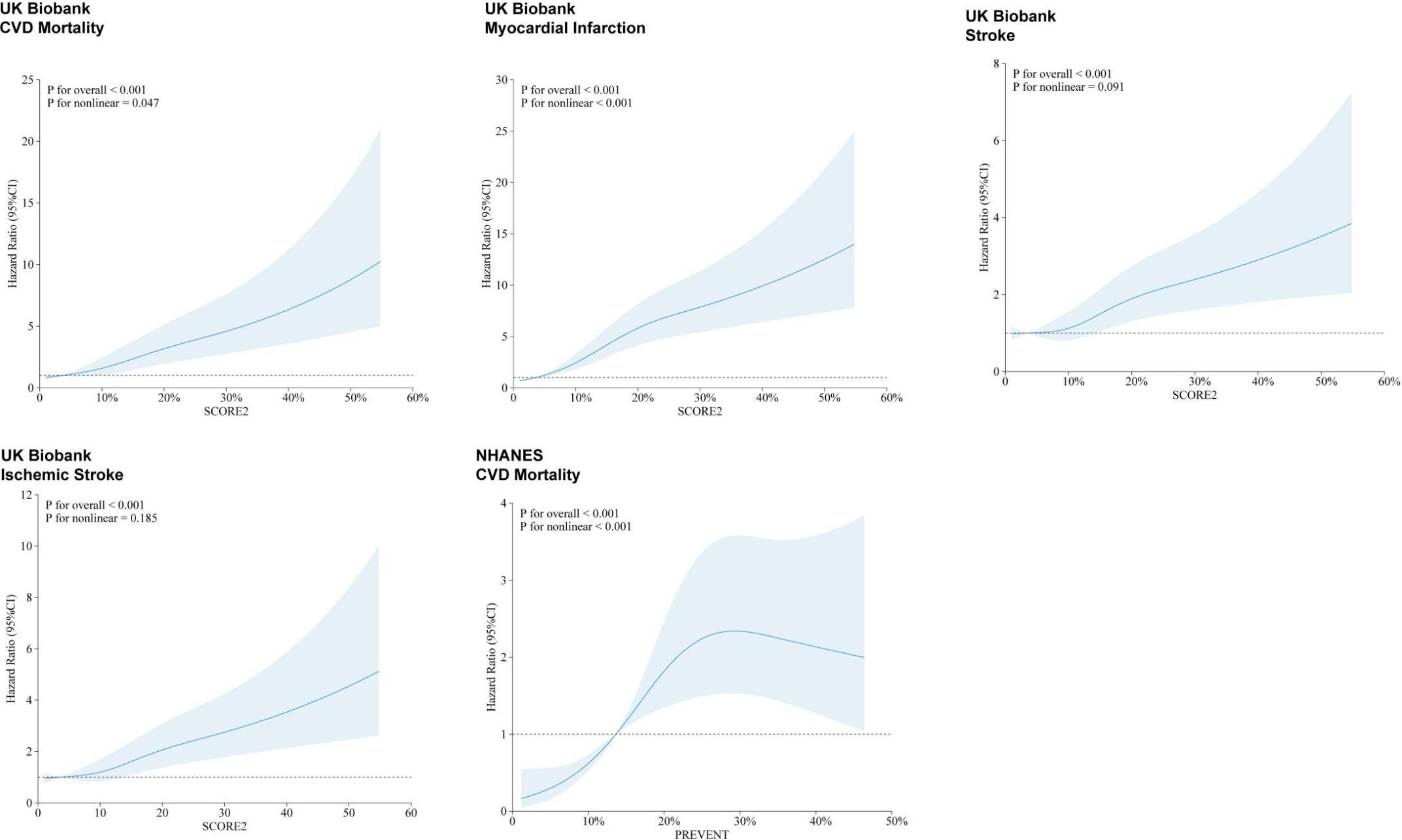


## **Figure S4.** Restricted cubic spline (RCS) curve illustrating the associations of SCORE2 and PREVENT equation with adverse cardiovascular events.

**Note:** UKBB: Model was adjusted by age (continuous), sex (male, female), ethnicity/race (White, Asian or Asian British, Black or Black British,

Chinese, Mixed, Other ethnic group), educational level (less than high school, high school and above), smoking status (Yes or No), alcohol consumption (continuous), physical activity (adequate, inadequate), TC (continuous), Townsend deprivation index (three categories stratified based on tertiles), history of diabetes.

**Abbreviations:** TyG=triglyceride-glucose, BMI=body mass index, WC=waist circumference, WHTR=waist circumference/height ratio, TG=triglyceride, HDL-C=High Density Lipoprotein-cholesterol, METSIR= Metabolic score for insulin resistance, CMI=cardiometabolic index, CVD=cardiovascular disease.


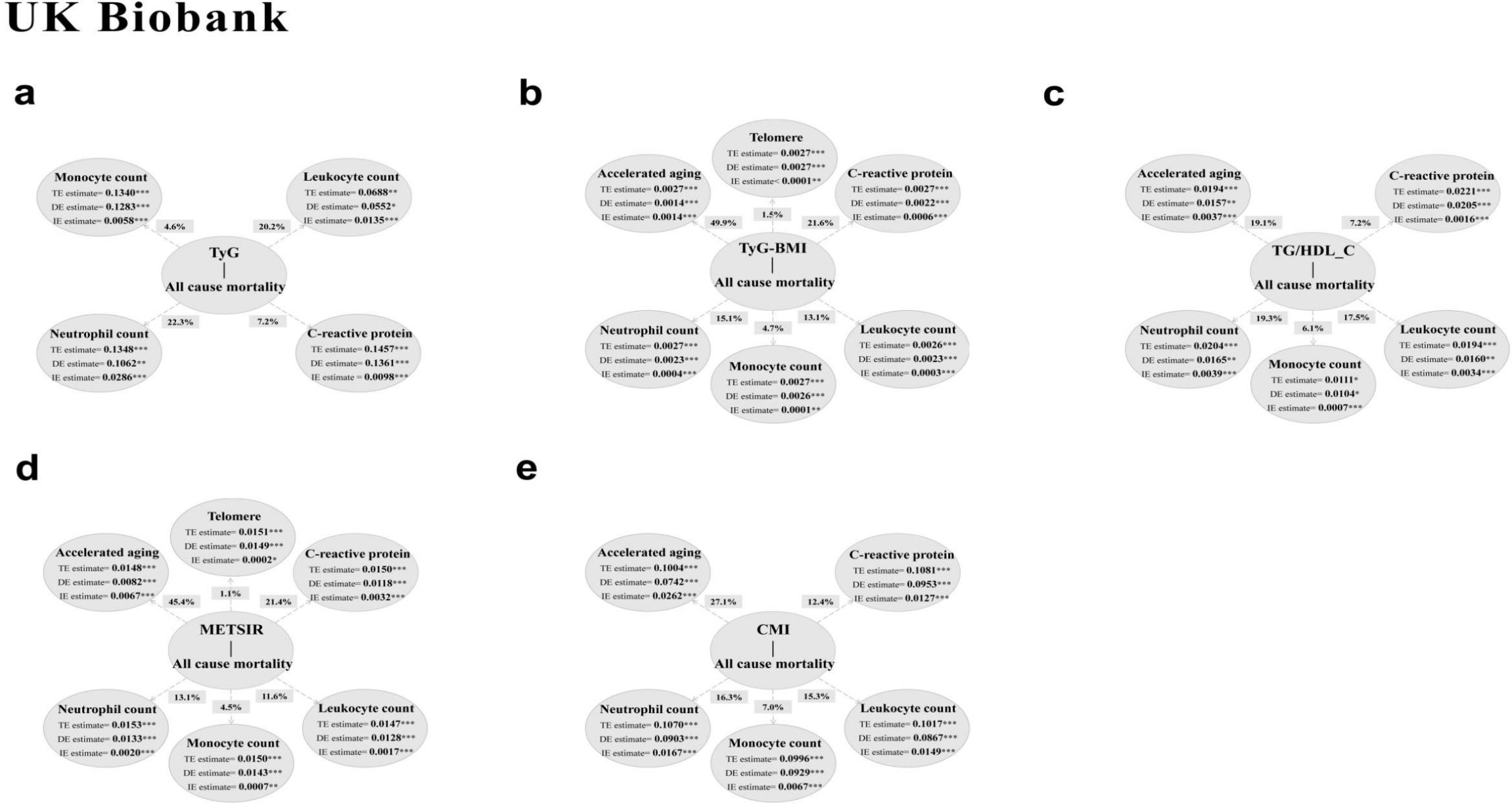


## **Figure S5.** Inflammatory markers and epigenetic aging mediate the associations of insulin resistance (IR)-related indices with adverse cardiovascular events in UK Biobank.

**Note:** Model was adjusted by age (continuous), sex (male, female), ethnicity/race (White, Asian or Asian British, Black or Black British,

Chinese, Mixed, Other ethnic group), educational level (less than high school, high school and above), smoking status (Yes or No), alcohol consumption (continuous), physical activity (adequate, inadequate), TC (continuous), Townsend deprivation index (three categories stratified based on tertiles), history of diabetes.

Abbreviations: TyG=triglyceride-glucose, BMI=body mass index, WC=waist circumference, WHTR=waist circumference/height ratio, TG=triglyceride, HDL-C=High Density Lipoprotein-cholesterol, METSIR= Metabolic score for insulin resistance, CMI=cardiometabolic index, CVD=cardiovascular disease.

# TyG TyG-BMI TyG-WC TyG-WHTR TG/HDL_C CMI METS_IR SCORE2/PREVENT

**UK Biobank CVD Mortality**


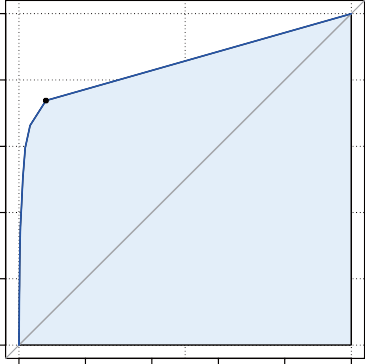


0.001 (0.919, 0.738)

AUC: 0.849


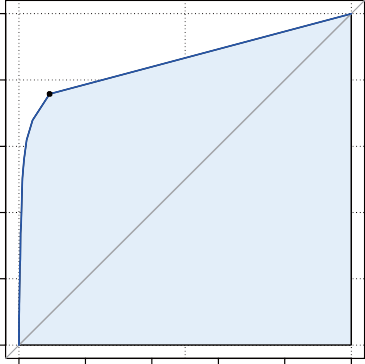


0.001 (0.908, 0.758)

AUC: 0.857


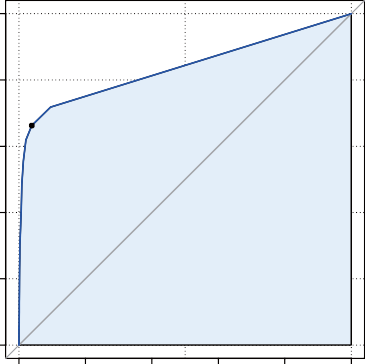


0.003 (0.962, 0.663)

AUC: 0.838


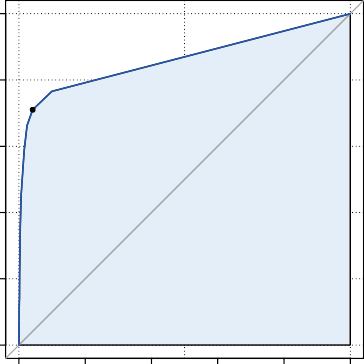


0.003 (0.959, 0.710)

AUC: 0.862


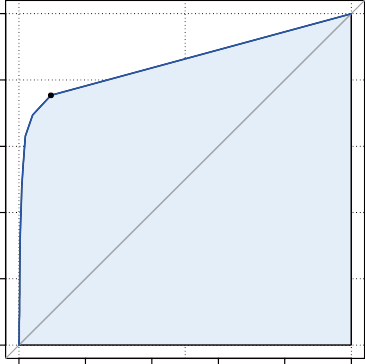


0.001 (0.904, 0.754)

AUC: 0.856


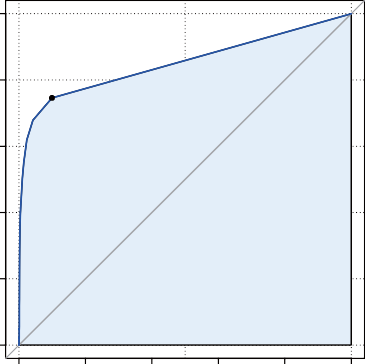


0.001 (0.901, 0.746)

AUC: 0.850


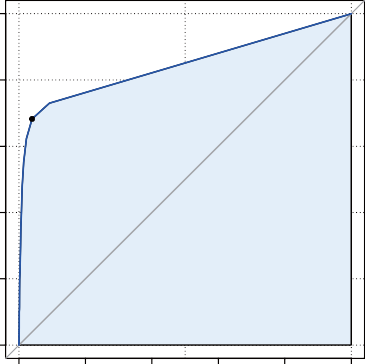


0.003 (0.961, 0.683)

AUC: 0.844


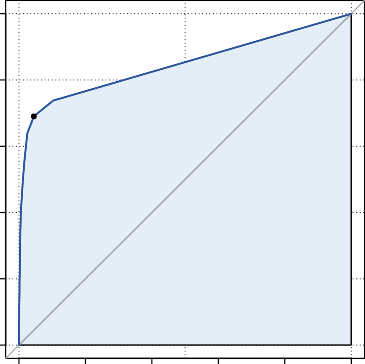


0.003 (0.955, 0.690)

AUC: 0.846

Sensitivity

0.2

0.4

0.6

0.8

1.0

Sensitivity

0.2

0.4

0.6

0.8

1.0

Sensitivity

0.2

0.4

0.6

0.8

1.0

Sensitivity

0.2

0.4

0.6

0.8

1.0

Sensitivity

0.2

0.4

0.6

0.8

1.0

Sensitivity

0.2

0.4

0.6

0.8

1.0

Sensitivity

0.2

0.4

0.6

0.8

1.0

Sensitivity

0.2

0.4

0.6

0.8

1.0

0.0 0.2 0.4 0.6 0.8 1.0

0.0

0.0

0.0

0.0

0.0

0.0

0.0

0.0

1 − Specificity

1.0

0.0 0.2 0.4 0.6 0.8 1.0

1 − Specificity

1.0

0.0 0.2 0.4 0.6 0.8 1.0

1 − Specificity

1.0

0.0 0.2 0.4 0.6 0.8 1.0

1 − Specificity

1.0

0.0 0.2 0.4 0.6 0.8 1.0

1 − Specificity

1.0

0.0 0.2 0.4 0.6 0.8 1.0

1 − Specificity

1.0

0.0 0.2 0.4 0.6 0.8 1.0

1 − Specificity

1.0

0.0 0.2 0.4 0.6 0.8 1.0

1 − Specificity

1.0

# UK Biobank Myocardial Infarction


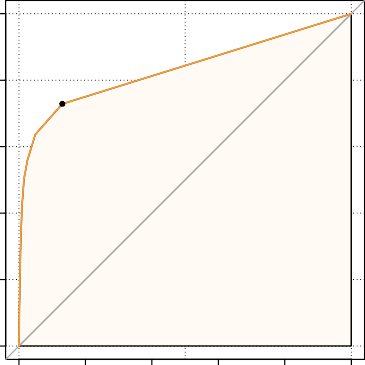


0.001 (0.870, 0.729)

AUC: 0.832


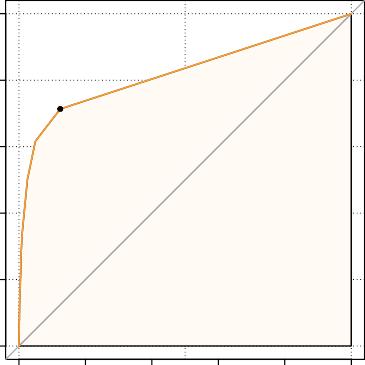


0.001 (0.876, 0.714)

AUC: 0.822


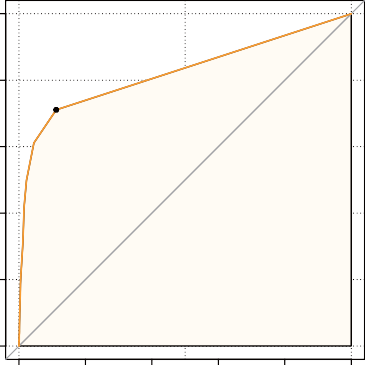


0.001 (0.888, 0.711)

AUC: 0.823


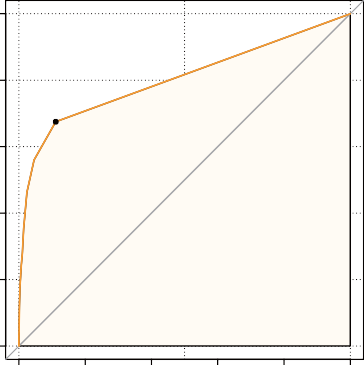


0.001 (0.889, 0.675)

AUC: 0.803


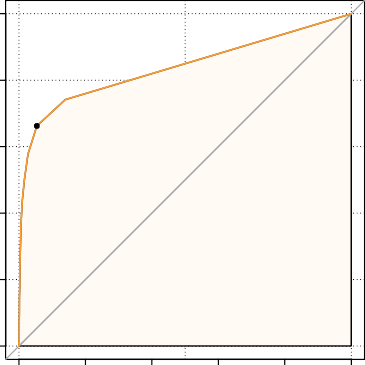


0.003 (0.946, 0.662)

AUC: 0.838


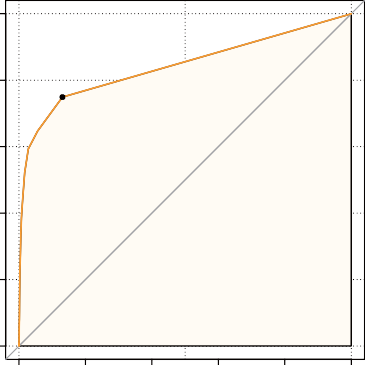


0.001 (0.869, 0.749)

AUC: 0.842


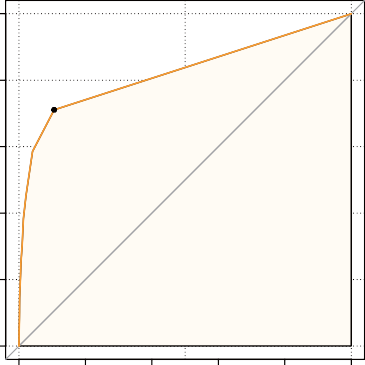


0.001 (0.894, 0.711)

AUC: 0.824


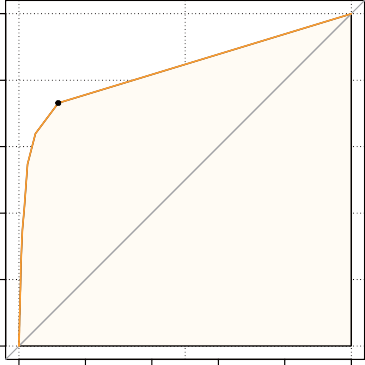


0.001 (0.882, 0.731)

AUC: 0.834

Sensitivity

0.2

0.4

0.6

0.8

Sensitivity

0.2

0.4

0.6

0.8

Sensitivity

0.2

0.4

0.6

0.8

Sensitivity

0.2

0.4

0.6

0.8

Sensitivity

0.2

0.4

0.6

0.8

Sensitivity

0.2

0.4

0.6

0.8

Sensitivity

0.2

0.4

0.6

0.8

Sensitivity

0.2

0.4

0.6

0.8

0.0 0.2 0.4 0.6 0.8 1.0

0.0

0.0

0.0

0.0

0.0

0.0

0.0

0.0

1 − Specificity

1.0

0.0 0.2 0.4 0.6 0.8 1.0

1 − Specificity

1.0

0.0 0.2 0.4 0.6 0.8 1.0

1 − Specificity

1.0

0.0 0.2 0.4 0.6 0.8 1.0

1 − Specificity

1.0

0.0 0.2 0.4 0.6 0.8 1.0

1 − Specificity

1.0

0.0 0.2 0.4 0.6 0.8 1.0

1 − Specificity

1.0

0.0 0.2 0.4 0.6 0.8 1.0

1 − Specificity

1.0

0.0 0.2 0.4 0.6 0.8 1.0

1 − Specificity

1.0

# UK Biobank Stroke


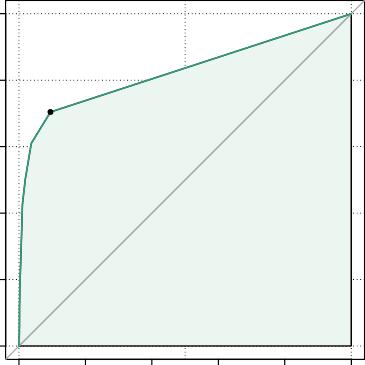


0.001 (0.905, 0.704)

AUC: 0.826


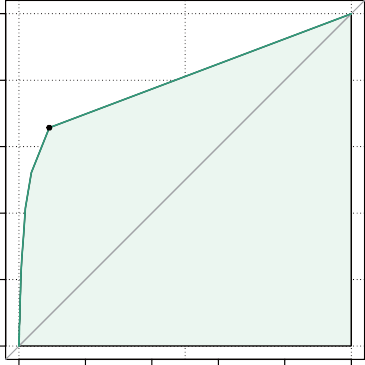


0.001 (0.909, 0.657)

AUC: 0.798


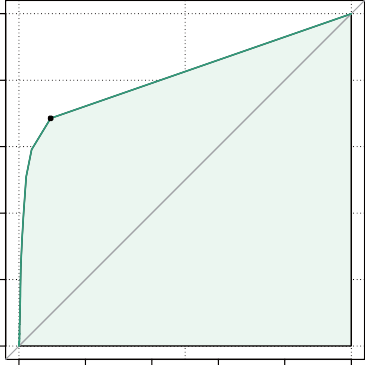


0.001 (0.905, 0.686)

AUC: 0.815


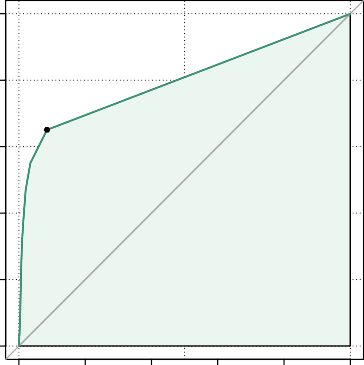


0.001 (0.915, 0.651)

AUC: 0.799


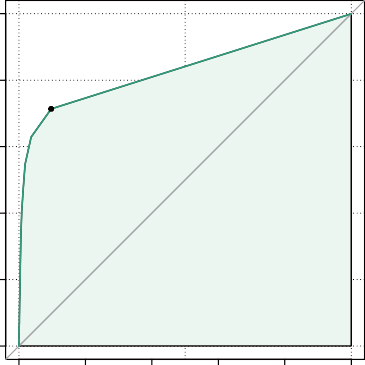


0.001 (0.903, 0.714)

AUC: 0.832


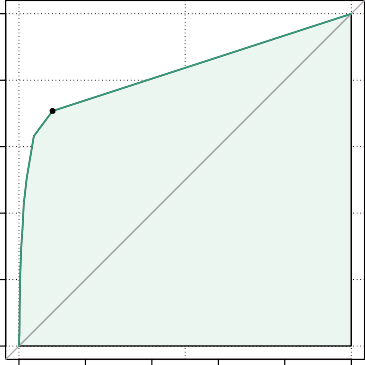


0.001 (0.899, 0.708)

AUC: 0.826


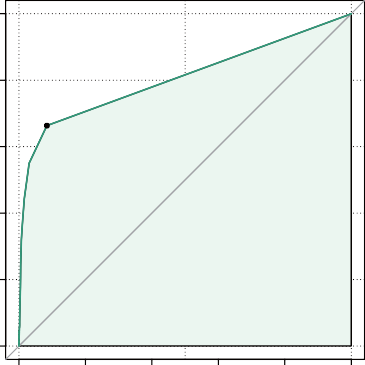


0.001 (0.916, 0.664)

AUC: 0.806


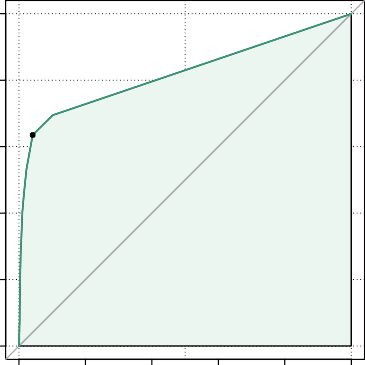


0.003 (0.959, 0.635)

AUC: 0.821

Sensitivity

0.2

0.4

0.6

0.8

Sensitivity

0.2

0.4

0.6

0.8

Sensitivity

0.2

0.4

0.6

0.8

Sensitivity

0.2

0.4

0.6

0.8

Sensitivity

0.2

0.4

0.6

0.8

Sensitivity

0.2

0.4

0.6

0.8

Sensitivity

0.2

0.4

0.6

0.8

Sensitivity

0.2

0.4

0.6

0.8

0.0 0.2 0.4 0.6 0.8 1.0

0.0

0.0

0.0

0.0

0.0

0.0

0.0

0.0

1 − Specificity

1.0

0.0 0.2 0.4 0.6 0.8 1.0

1 − Specificity

1.0

0.0 0.2 0.4 0.6 0.8 1.0

1 − Specificity

1.0

0.0 0.2 0.4 0.6 0.8 1.0

1 − Specificity

1.0

0.0 0.2 0.4 0.6 0.8 1.0

1 − Specificity

1.0

0.0 0.2 0.4 0.6 0.8 1.0

1 − Specificity

1.0

0.0 0.2 0.4 0.6 0.8 1.0

1 − Specificity

1.0

0.0 0.2 0.4 0.6 0.8 1.0

1 − Specificity

1.0

# UK Biobank Ischemic Stroke


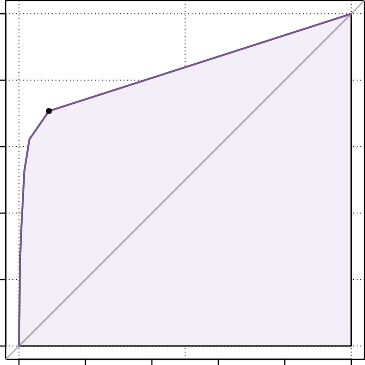


0.001 (0.910, 0.707)

AUC: 0.830


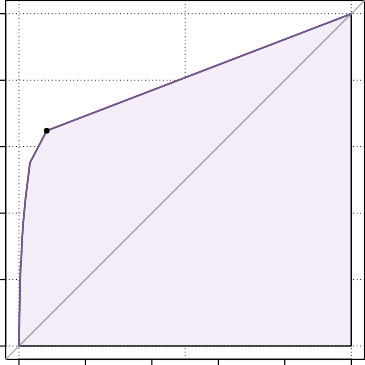


0.001 (0.917, 0.648)

AUC: 0.798


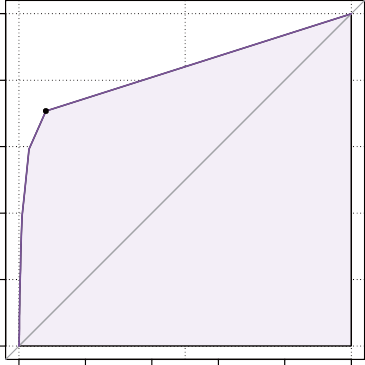


0.001 (0.919, 0.707)

AUC: 0.830


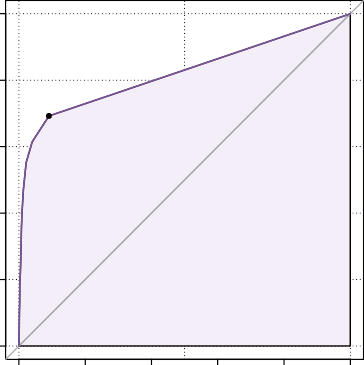


0.001 (0.909, 0.693)

AUC: 0.822


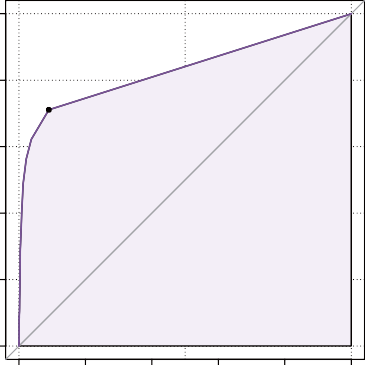


0.001 (0.910, 0.711)

AUC: 0.832


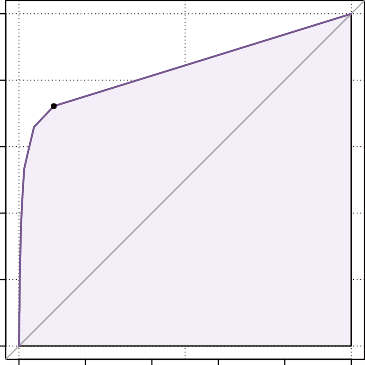


0.001 (0.895, 0.722)

AUC: 0.835


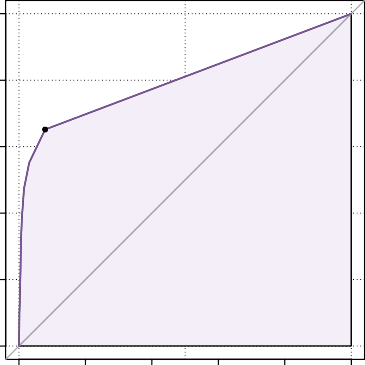


0.001 (0.921, 0.652)

AUC: 0.803


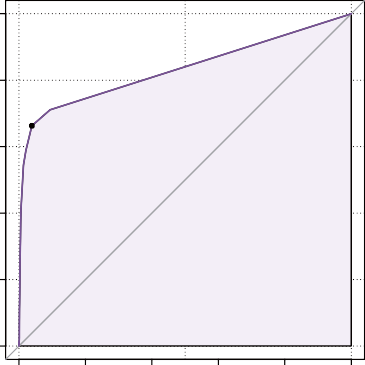


0.003 (0.961, 0.663)

AUC: 0.833

Sensitivity

0.2

0.4

0.6

0.8

Sensitivity

0.2

0.4

0.6

0.8

Sensitivity

0.2

0.4

0.6

0.8

Sensitivity

0.2

0.4

0.6

0.8

Sensitivity

0.2

0.4

0.6

0.8

Sensitivity

0.2

0.4

0.6

0.8

Sensitivity

0.2

0.4

0.6

0.8

Sensitivity

0.2

0.4

0.6

0.8

0.0 0.2 0.4 0.6 0.8 1.0

0.0

0.0

0.0

0.0

0.0

0.0

0.0

0.0

1 − Specificity

0.0 0.2 0.4 0.6 0.8 1.0

1 − Specificity

0.0 0.2 0.4 0.6 0.8 1.0

1 − Specificity

0.0 0.2 0.4 0.6 0.8 1.0

1 − Specificity

1.

0.0 0.2 0.4 0.6 0.8 1.0

1 − Specificity

1.0

0.0 0.2 0.4 0.6 0.8 1.0

1 − Specificity

1.0

0.0 0.2 0.4 0.6 0.8 1.0

1 − Specificity

1.0

0.0 0.2 0.4 0.6 0.8 1.0

1 − Specificity

1.0

## **Figure S6.** Random forest plot evaluating the prediction accuracy of insulin resistance (IR)-related indices, SCORE2 and PREVENT in random forest plots.

**Note:** Model was adjusted by age (continuous), sex (male, female).

Abbreviations: TyG=triglyceride-glucose, BMI=body mass index, WC=waist circumference, WHTR=waist circumference/height ratio, TG=triglyceride, HDL-C=High Density Lipoprotein-cholesterol, METSIR=Metabolic score for insulin resistance, CMI=cardiometabolic index, CVD=cardiovascular disease.


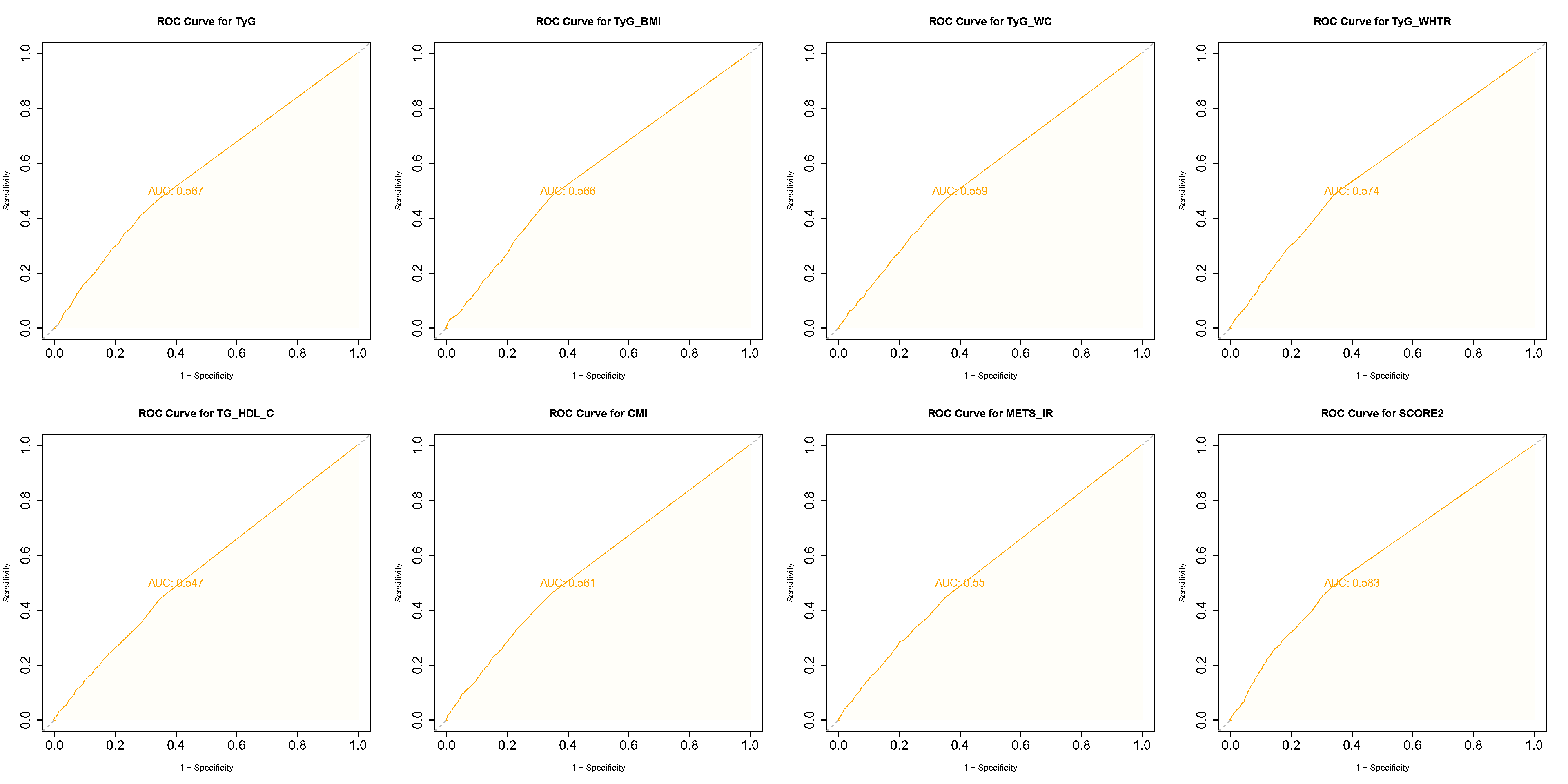


**Figure S7.** ROC Curves from 5-Fold Cross-Validation of Random Forest Models.

**Note:** Model was adjusted by age (continuous), sex (male, female).

Abbreviations: TyG=triglyceride-glucose, BMI=body mass index, WC=waist circumference, WHTR=waist circumference/height ratio, TG=triglyceride, HDL-C=High Density Lipoprotein-cholesterol, METSIR=Metabolic score for insulin resistance, CMI=cardiometabolic index, CVD=cardiovascular disease.
